# Supplementary material for: Contributions of different host species to the natural transmission of severe fever with thrombocytopenia syndrome virus in China
Source: PLoS Negl Trop Dis. 2025 Jul 17;19(7):e0013304. doi: 10.1371/journal.pntd.0013304 (PMC12286343; doi:10.1371/journal.pntd.0013304)
Supplement: S1 Text — (DOCX) [file pntd.0013304.s001.docx]

**S1 Text. Multi-host mathematical model**

Based on their disease status, the total population $N_{i}$ of each host species *i* was partitioned into four subpopulations: the susceptible ($S_{i})$, exposed ($E_{i})$, infectious ($I_{i})$, and recovered ($R_{i})$ individuals. The tick population $N_{T}$ was partitioned into two subpopulations: the susceptible ($S_{T})$ and infectious ($I_{T})$ ticks. For simplicity, we did not differentiate between the various life stages of ticks (i.e., eggs, larva, nymphs and adults). We assumed closed and stationery populations for all host species and the ticks, where their mortality rates equaled birth rates ($\mu_{i}$ for host species *i* and $\mu_{T}$ for tick). SFTSV is transmitted between host animals and ticks through tick bites. We assumed different contact rates with ticks for different host species ($\beta_{i}$ for host species i) due to the apparent host preferences of *H. longicornis* [1]. Once bitten, individuals transitioned from $E_{i}$ to $I_{i}$ at a rate of $\gamma_{i}$, with $1/\gamma_{i}$ equals the average duration of the latent period. We assumed an excessive SFTS-induced mortality rate of $\rho_{i}$ for host species *i*, while no excessive mortality rate for the ticks [2, 3]. The durations of the infectious period ${1/\sigma}_{i}$ varied widely between species, ranging from one day in goats [4] to ten days in hedgehogs [5]. Transovarial transmission of SFTSV was included in the model through attributing a proportion $\phi$ of the offspring of infectious ticks directly to the $I_{T}$ subgroup.

**State variables**

*Host species*

$S_{i}$ – the proportion of individuals in species *i* that are susceptible

$E_{i}$ – the proportion of individuals in species *i* that are exposed to SFTSV

$I_{i}$ – the proportion of individuals in species *i* that are infectious

$R_{i}$ – the proportion of individuals in species *i* that are recovered

*Tick species*

$S_{T}$ – the proportion of ticks that are susceptible

$I_{T}$ – the proportion of ticks that are infectious

**Initial values**

The initial values of $S_{i}$, $E_{i}$, $I_{i}$ and $R_{i}$ were set to $1-{10}^{-6}, 0, {10}^{-6},$ and 0, respectively, and those for $S_{T}$ and $I_{T}$ were set to 1 and 0, respectively. Since we ran the model to equilibrium, the initial values have no impacts on the estimation in each subpopulation.

**Differential equations**

*For host species i*

$\frac{dS_{i}}{dt}=\mu_{i}-\beta_{i}I_{T}S_{i}-\mu_{i}S_{i}$

$\frac{dE_{i}}{dt}=\beta_{i}I_{T}S_{i}-\gamma_{i}E_{i}-\mu_{i}E_{i}$

$\frac{dI_{i}}{dt}=\gamma_{i}E_{i}-\sigma_{i}I_{i}-\rho_{i}I_{i}-\mu_{i}I_{i}$ (Eq. 1)

$\frac{dR_{i}}{dt}=\sigma_{i}I_{i}-\mu_{i}R_{i}$

*For ticks*

$\frac{dS_{T}}{dt}=\left( 1-\phi\right)\mu_{T}I_{T}-\Sigma\beta_{i}\chi_{i}I_{i}S_{T}$

$\frac{dI_{T}}{dt}=\phi\mu_{T}I_{T}+\Sigma\beta_{i}\chi_{i}I_{i}S_{T}-\mu_{T}I_{T}$

We ran the model for 10000 days to achieve equilibrium. All analyses were performed in R 4.2.3, with packages odin 1.2.4 for developing the multiple-host mathematical model, doSNOW 1.0.20 and doParallel 1.0.17 for parallel computation, Hmisc 4.7-0 for the Wilson score interval method, tidyverse 1.3.2 for data processing, and ggplot2 3.4.3, cowplot 1.1.1, ggmap 3.0.2, ggrepel 0.9.1, ggthemes 4.2.4, ggsci 2.9, packcircles 0.3.6, ggraph 2.1.0, and ggridges 0.5.4 for visualization. All the code and data are available at: <https://github.com/qu-cheng/SFTS_host>.

**References**

1. Tian J, Wu T, Chen X, Bao J, Xu R, Zhang Y. The distribution of ticks in Wuhan and surrounding ares. Acta Parasitologica et Medica Entomologica Sinica,. 2014;(2):129-33.

2. Hu Y. Study on the transmission of severe fever with thrombocytopenia syndrome virus in different tick species. Beijing: Beijing Institute of Microbiology and Epidemiology; 2020.

3. Zhuang L. Pathogens in ticks and experimental study on maintenance and transmission of SFTSV by Haemaphysalis longicornis. Beijing: Beijing Institute of Microbiology and Epidemiology; 2014.

4. Jiao Y, Qi X, Liu D, Zeng X, Han Y, Guo X, et al. Experimental and Natural Infections of Goats with Severe Fever with Thrombocytopenia Syndrome Virus: Evidence for Ticks as Viral Vector. PLOS Neglected Tropical Diseases. 2015;9(10):e0004092. doi: 10.1371/journal.pntd.0004092.

5. Zhao C, Zhang X, Si X, Ye L, Lawrence K, Lu Y, et al. Hedgehogs as Amplifying Hosts of Severe Fever with Thrombocytopenia Syndrome Virus, China. Emerging Infectious Diseases. 2022;28(12):2491.
