# Supplementary material for: Contributions of different host species to the natural transmission of severe fever with thrombocytopenia syndrome virus in China
Source: PLoS Negl Trop Dis. 2025 Jul 17;19(7):e0013304. doi: 10.1371/journal.pntd.0013304 (PMC12286343; doi:10.1371/journal.pntd.0013304)
Supplement: S2 Text — (DOCX) [file pntd.0013304.s002.docx]

**S2 Text. Model calibration**

First, we randomly drew parameter sets from the ranges presented in Table 1 and ran the model using these values until equilibrium was reached. Next, we compared $R_{i}^{*}$, the steady state value for $R_{i}$, with the confidence interval of the surveyed seroprevalence rate for species *i*. We made this comparison based on the assumption that after infection, the total antibodies (including IgG and IgM) and immunity of the host animals to SFTSV lasted a long time [1-3]. Parameter sets yielding $R_{i}^{*}$ within the confidence interval for all species were defined as passing parameter sets. We continued sampling and simulating until a total of 1,000 passes were collected. We further used the sampling-importance resampling approach to account for the differences in the goodness-of-fit of these 1,000 passes, where we sampled 10,000 times from these 1,000 samples with replacement, weighting by their likelihoods of producing the observed seroprevalence rates [4, 5]. These 10,000 parameter sets were then utilized to estimate the overall basic reproduction number $R_{0}$ and species-level $R_{0i}$.

More specifically, we calibrated a total of 4*k*+3 parameters for a location with k surveyed species, including $\beta_{i}$, $\gamma_{i}$, $\upsilon_{i}$, and $\sigma_{i}$ for each species, as well as $\mu_{T}$, $\phi$, and $\chi_{max}$. We first drew random values from the ranges shown in Table for these parameters, then used the sampled $\upsilon_{i}$ and $\sigma_{i}$ to calculate the model parameter $\rho_{i}$ as $\rho_{i}=\frac{\upsilon_{i}(\mu_{i}+\sigma_{i})}{1-\upsilon_{i}}$ (since $\upsilon_{i}=\frac{\rho_{i}}{\rho_{i}+\mu_{i}+\sigma_{i}}$), and used the sampled $\chi_{max}$ to calculate $\chi_{i}$ as $\chi_{i}=\frac{\chi_{max}N_{i}}{N_{max}}$, where $N_{i}$ and $N_{max}$ represent the abundance for the species *i* and the abundance for the most abundant species, respectively. $N_{i}$ was either directly collected from statistical yearbooks or inferred from the density reported in literature (Table ST3.1).

**Table ST3.1** Methods for estimating the abundance of each host species

| **Species** | **Abundance** | **Refences** | |
| --- | --- | --- | --- |
| Goat/sheep, cattle, pig, poultry | From statistical yearbooks | [2, 6-17] |  |
| Dog | Number of human households/10 | [18] |  |
| Rodent | Human population size * 2 | [19] |  |
| Hedgehog | Area * 80 per square kilometer | [20] |  |
| Hare | Area * 29.8 per square kilometer | [21] |  |
| Yellow weasel | Area * 2.85 per square kilometer | [22, 23] |  |
| Wild bird | Green space area * 400 per square kilometer | [24, 25] |  |

Due to the extremely low passing rate in the original parameter space (usually only a few passes in one million simulations, where a passing parameter set is defined as a set of parameters that produces $R_{i}^{*}$s, the steady state value for $R_{i}$s, within the confidence intervals of observed seroprevalence rates for all species), we trimmed the ranges of $\beta_{i}$s and $\chi_{max}$ multiple rounds to remove the parameter space with no passes, thereby increasing the passing rate in the remaining parameter space. We chose to focus only on these two parameters since the preliminary data analysis suggested that they were the most important in determining $R_{0i}$.

In Round 1, we ran the model a million times and filtered out the partial passing parameter sets that resulted in $R_{i}^{*}$s matching the surveyed seroprevalence rates (i.e., fell within their confidence interval) for at least two species (hereinafter referred to as “passing species”). These partial passing parameter sets can be used to refine the ranges of $\beta_{i}$s for the next round. Specifically, we first tallied the number of parameter sets retained in Round 1 for each combination (e.g., pair, triplet, quadruplet, etc.) of passing species. Then, for each combination with over 100 parameter sets, we obtained their minimum values $\beta_{individual, min\_i}$and maximum values $\beta_{individual, max\_i}$. We did not utilize data from the combinations with fewer than 100 partial passing parameter sets since they may not be able to adequately represent the true parameter distributions due to their small sample sizes. The maximum value of $\beta_{individual, min\_i}$ and the minimum value of $\beta_{individual, max\_i}$across combinations were then employed to define the new ranges for $\beta_{i}$s in the next round as [0.8*$max(\beta_{individual, min\_i})$, min(1.2*min($\beta_{individual, max\_i})$, 2)].

In Round 2, we sampled from the updated ranges (R2 ranges) and ran the model for a million times. We continued to use these ranges when the passing rate was sufficiently in this round, while trimmed the ranges again for the next round when the passing rate was low. First, we counted the number of passes (i.e., matching the surveyed seroprevalence rates for all species) in the one million runs. In cases where more than 1,000 passes were obtained, we stopped and took the first 1,000 as our final passes. In cases where the number of passes was between 801 and 1,000, we continued sampling from R2 ranges, until we got 1,000 final passes. In cases where the number of passes ranged from 50 to 800, we saved the passes and used them to determine the parameter ranges for Round 3 following the method described below. In cases where the number of passes was insufficient to provide reliable information (i.e., fewer than 50 passes), we continued sampling from R2 ranges until we acquired at least 50 passes. These passes were used to determine the range of $\beta_{max}\chi_{max}$ for Round 3. We chose to pose constraint on $\beta_{max}\chi_{max}$, since the preliminary data analysis suggested that the product of $\beta_{max}$ and $\chi_{max}$ of the passing parameter sets had an upper bound. We obtained the maximum value of $\beta_{max}\chi_{max}$ from the passes, and set the upper bound of $\beta_{max}\chi_{max}$ in R3 ranges to be 1.2 times this maximum value.

In Round 3, we kept sampling from the R3 ranges, until we got at least 1,000 passes.

**References**

1. Ishijima K, Tatemoto K, Park E, Kimura M, Fujita O, Taira M, et al. Lethal disease in dogs naturally infected with severe fever with thrombocytopenia syndrome virus. Viruses. 2022;14(9):1963.

2. Niu G, Li J, Liang M, Jiang X, Jiang M, Yin H, et al. Severe fever with thrombocytopenia syndrome virus among domesticated animals, China. Emerging infectious diseases. 2013;19(5):756.

3. Li A, Dai X, Chen L, Liu L, Li C, Liu Y, et al. Immunogenicity and protective efficacy of an inactivated SFTS vaccine candidate in mice. Biosafety and Health. 2022;4(01):45-52.

4. Brouwer AF, Eisenberg MC, Bakker KM, Boerger SN, Zahid MH, Freeman MC, et al. Leveraging infectious disease models to interpret randomized controlled trials: Controlling enteric pathogen transmission through water, sanitation, and hygiene interventions. PLOS Computational Biology. 2022;18(12):e1010748.

5. Smith AF, Gelfand AE. Bayesian statistics without tears: A sampling-resampling perspective. American statistician. 1992:84-8.

6. Wuxi SBo. Wuxi statistical Yearbook, 2011. Beijing: China Statistics Press.

7. Wuxi SBo. Wuxi statistical Yearbook, 2013. Beijing: China Statistics Press.

8. Statis, Changzhou tBo. Changzhou statistical Yearbook, 2011. Beijing: China Statistics Press.

9. Changzhou SBo. Changzhou statistical Yearbook, 2013. Beijing: China Statistics Press.

10. Nanjing SBo. Nanjing statistical Yearbook, 2011. Phoenix Press.

11. Nanjing SBo. Nanjing statistical Yearbook, 2013. Beijing: China Statistics Press.

12. Huaian SBo. Huaian statistical Yearbook, 2012. Beijing: China Statistics Press.

13. Huaian SBo. Huaian statistical Yearbook, 2010. Statistics Bureau of Huaian

14. Lianyungang SBo. Lianyungang statistical Yearbook, 2011. Beijing: China Statistics Press.

15. Lianyungang SBo. Lianyungang statistical Yearbook, 2013. Beijing: China Statistics Press.

16. Xinyang SBo. Xinyang statistical Yearbook, 2013.

17. Xinyang SBo. Xinyang statistical Yearbook, 2018. Beijing: China Statistics Press.

18. Beijing Nuotuo Information Consulting Co. L. Pet Industry Market In-depth Analysis and Investment Prospects Research Report.

19. Dodwell D. How many rats are there in Hong Kong? Their thriving population reflects human society’s inability to clean up after ourselves. 2019.

20. Zhao C, Zhang X, Si X, Ye L, Lawrence K, Lu Y, et al. Hedgehogs as Amplifying Hosts of Severe Fever with Thrombocytopenia Syndrome Virus, China. Emerging Infectious Diseases. 2022;28(12):2491.

21. Fan Y-Y. Cape Hare's Harm Research in Western Liaoning [Master]: Northeast Forestry University; 2009.

22. Piao Z, Tang L, Swihart RK, Wang S. Human–wildlife competition for Korean pine seeds: vertebrate responses and implications for mixed forests on Changbai Mountain, China. Annals of Forest Science. 2011;68(5):p.911-9.

23. Sheng H-L, Lu H-J. The environment preference of nesting and nest density of the female weaseis. Acta Theriologica Sinica. 1982;(01):29-34. doi: 10.16829/j.slxb.1982.01.007.

24. Teng W, Deng W. Population density and community characteristics of cavity-nesting birds in Beijing. Journal of Beijing Normal University (Natural Science). 2014;50(3):278-81.

25. Shi C, Yang G. A New Bird Record in Hohhot: Spotted Dove Streptopelia chinensis. Zoological research. 2006;27(6):656.
