# Supplementary material for: Contributions of different host species to the natural transmission of severe fever with thrombocytopenia syndrome virus in China
Source: PLoS Negl Trop Dis. 2025 Jul 17;19(7):e0013304. doi: 10.1371/journal.pntd.0013304 (PMC12286343; doi:10.1371/journal.pntd.0013304)
Supplement: S3 Text — (DOCX) [file pntd.0013304.s003.docx]

**S3 Text. Estimation of the basic reproduction numbers**

We first estimated the overall $R_{0}$ with the next-generation matrix method [1]. The infected subpopulations are $(E_{1}, I_{1}, E_{2}, I_{2},\ldots, E_{k}, I_{k}, I_{T})$, where k is the number of species being survyed. According to Eq. 1 in S1 Text, the transmission matrix $\boldsymbol{T}$ can be written as:

$\boldsymbol{T}=\left( \begin{matrix} 0 & 0 & 0 & 0 & \ldots& 0 & 0 & \beta_{1} \\ 0 & 0 & 0 & 0 & \ldots& 0 & 0 & 0 \\ 0 & 0 & 0 & 0 & \ldots& 0 & 0 & \beta_{2} \\ 0 & 0 & 0 & 0 & \ldots& 0 & 0 & 0 \\ \ldots& \ldots& \ldots& \ldots& \ldots& \ldots& \ldots& \ldots\\ 0 & 0 & 0 & 0 & \ldots& 0 & 0 & \beta_{k} \\ 0 & 0 & 0 & 0 & \ldots& 0 & 0 & 0 \\ 0 & \beta_{1}\chi_{1} & 0 & \beta_{2}\chi_{2} & \ldots& 0 & \beta_{k}\chi_{k} & \phi\mu_{T} \end{matrix} \right)$,

where $T_{ij}$, the element on the *i*th row and *j*th column of $\boldsymbol{T}$, represents the infection rate of individuals in subpopulation *i* resulted from individuals in subpopulation *j*. While the transition matrix $\boldsymbol{\Sigma}$can be written as:

$\boldsymbol{\Sigma}=\left( \begin{matrix} -(\gamma_{1}+\mu_{1}) & 0 & 0 & 0 & \ldots& 0 & 0 & 0 \\ \gamma_{1} & -(\sigma_{1}+\rho_{1}+\mu_{1}) & 0 & 0 & \ldots& 0 & 0 & 0 \\ 0 & 0 & -(\gamma_{2}+\mu_{2}) & 0 & \ldots& 0 & 0 & 0 \\ 0 & 0 & \gamma_{2} & -(\sigma_{2}+\rho_{2}+\mu_{2}) & \ldots& 0 & 0 & 0 \\ \ldots& \ldots& \ldots& \ldots& \ldots& \ldots& \ldots& \ldots\\ 0 & 0 & 0 & 0 & \ldots& -(\gamma_{k}+\mu_{k}) & 0 & 0 \\ 0 & 0 & 0 & 0 & \ldots& \gamma_{k} & -(\sigma_{k}+\rho_{k}+\mu_{k}) & 0 \\ 0 & 0 & 0 & 0 & \ldots& 0 & 0 & -\mu_{T} \end{matrix} \right)$, where $\Sigma_{ij}$ represents the change rate from subpopulation *j* to subpopulation *i* that does not involve the infection process. The next-generation matrix

$\boldsymbol{K}=-\boldsymbol{E}^{'}\boldsymbol{T}\boldsymbol{\Sigma}^{\boldsymbol{-1}}\boldsymbol{E}=\left( \begin{matrix} 0 & 0 & \ldots& 0 & \frac{\beta_{1}}{\mu_{T}} \\ 0 & 0 & \ldots& 0 & \frac{\beta_{2}}{\mu_{T}} \\ \ldots& \ldots& \ldots& \ldots& \ldots\\ 0 & 0 & \ldots& 0 & \frac{\beta_{k}}{\mu_{T}} \\ \frac{\beta_{1}\chi_{1}\gamma_{1}}{{(\gamma}_{1}+\mu_{1})(\sigma_{1}+\rho_{1}+\mu_{1})} & \frac{\beta_{2}\chi_{2}\gamma_{2}}{{(\gamma}_{2}+\mu_{2})(\sigma_{2}+\rho_{2}+\mu_{2})} & \ldots& \frac{\beta_{k}\chi_{k}\gamma_{k}}{{(\gamma}_{k}+\mu_{k})(\sigma_{k}+\rho_{k}+\mu_{k})} & \phi\end{matrix} \right)$,

where $\boldsymbol{E}=\left( \begin{matrix} 1 & 0 & \ldots& 0 & 0 \\ 0 & 0 & \ldots& 0 & 0 \\ 0 & 1 & \ldots& 0 & 0 \\ \ldots& \ldots& \ldots& \ldots& 0 \\ 0 & 0 & \ldots& 1 & 0 \\ 0 & 0 & \ldots& 0 & 1 \end{matrix} \right)$ is an auxiliary matrix to remove rows with only 0s in $\boldsymbol{T}$. The overall basic reproduction rate R_0_ can be estimated as the dominant eigen value of $\boldsymbol{K}$, with $R_{0}= \frac{\phi+\sqrt{\phi^{2}+\Sigma\frac{4\beta_{i}^{2}\chi_{i}\gamma_{i}}{\mu_{T}(\mu_{i}+\gamma_{i})(\mu_{i}+\rho_{i}+\sigma_{i})}}}{2}$.

The species-specific $R_{0i}$ was estimated following [2] as the dominant eigen value of $\left( \boldsymbol{P}_{\boldsymbol{H}_{\boldsymbol{i}}}+\boldsymbol{P}_{\boldsymbol{T}} \right)\boldsymbol{K}$, where $\boldsymbol{P}_{\boldsymbol{H}_{\boldsymbol{i}}}$ and $\boldsymbol{P}_{\boldsymbol{T}}$ are projection matrices to extract the elements related to host species *i* or ticks from **K**, respectively. $\boldsymbol{P}_{\boldsymbol{H}_{\boldsymbol{i}}}$ and $\boldsymbol{P}_{\boldsymbol{T}}$ both have the dimension of (k+1) ×(k+1), with the first k rows and columns represent animal hosts and the last row and column represent ticks. All elements of $\boldsymbol{P}_{\boldsymbol{H}_{\boldsymbol{i}}}$ are 0, except $\boldsymbol{P}_{\boldsymbol{H}_{\boldsymbol{i}}}\boldsymbol{[}i,i\boldsymbol{]}$, which is set to 1, while all elements of $\boldsymbol{P}_{\boldsymbol{T}}$ are set to 0, except $\boldsymbol{P}_{\boldsymbol{T}}\boldsymbol{[}k+1\boldsymbol{,}k+1\boldsymbol{]}$, which is set to 1. Here $A\boldsymbol{[}i,j\boldsymbol{]}$ means the element at row $i$ and column $j$ of matrix $\boldsymbol{A}$. The species-specific $R_{0i}$ can be written as $R_{0i}= \frac{\phi+\sqrt{\phi^{2}+\frac{4\beta_{i}^{2}\chi_{i}\gamma_{i}}{\mu_{T}(\mu_{i}+\gamma_{i})(\mu_{i}+\rho_{i}+\sigma_{i})}}}{2}$. Note that when there is no transvarial transmission in the ticks ($\phi=0)$, $R_{0}$ equals the sum of all $R_{0i}$s, which is $R_{0}=\sum_{i=0}^{k} R_{0i}$.

## **References**

1. Diekmann O, Heesterbeek J, Roberts MG. The construction of next-generation matrices for compartmental epidemic models. Journal of the royal society interface. 2010;7(47):873-85.

2. Funk S, Nishiura H, Heesterbeek H, Edmunds WJ, Checchi F. Identifying transmission cycles at the human-animal interface: the role of animal reservoirs in maintaining gambiense human african trypanosomiasis. PLoS computational biology. 2013;9(1):e1002855.
