# Supplementary material for: Contributions of different host species to the natural transmission of severe fever with thrombocytopenia syndrome virus in China
Source: PLoS Negl Trop Dis. 2025 Jul 17;19(7):e0013304. doi: 10.1371/journal.pntd.0013304 (PMC12286343; doi:10.1371/journal.pntd.0013304)
Supplement: S1 Table — The numbers in the parentheses are the 95% CI. The most important species for each survey are highlighted in bold. (DOCX) [file pntd.0013304.s004.docx]

**Table S1. Basic information, estimated overall** $\boldsymbol{R}_{\mathbf{0}}$**, and species-level** $\boldsymbol{R}_{\mathbf{0i}}$**s of the included seroprevalence surveys.** The numbers in the parentheses are the 95% CI. The most important species for each survey are highlighted in bold.

| **Survey ID and ref.** | **Locations** | **Years of the survey** | **Overall** $\boldsymbol{R}_{\mathbf{0}}$ | **Host species** | **Prevalence rate %** | **Species-level** $\boldsymbol{R}_{\mathbf{0i}}$ | **Reduction in overall R_0_ (%) after halving** $\boldsymbol{\beta}_{\boldsymbol{i}}$ |
| --- | --- | --- | --- | --- | --- | --- | --- |
| 1 (32) | Yixing, Liyang, Lishui, Jiangning, Xuyi, and Donghai Counties of Jiangsu Province | 2010 | 1.21 (1.04, 1.39) | **Goat/sheep** | **57.65 (47.04, 67.6)** | **1.13 (0.90, 1.35)** | **21.5** |
|  |  |  |  | Cattle | 32.22 (23.47, 42.43) | 0.55 (0.11, 0.93) | 0.3 |
|  |  |  |  | Poultry | 0.84 (0.28, 2.43) | 0.59 (0.13, 0.94) | 1.2 |
|  |  |  |  | Dog | 6.40 (3.28, 12.12) | 0.54 (0.06, 0.93) | 0 |
|  |  |  |  | Pig | 5.33 (3.08, 9.09) | 0.60 (0.20, 0.94) | 1.7 |
|  |  |  |  | Rodent | 0.00 (0.00, 7.56) | 0.64 (0.14, 0.95) | 4.3 |
| 2 (7) | Laizhou Prefecture, Shandong Province | 2011 | 1.48 (1.17, 1.63) | Goat/sheep | 69.27 (62.65, 75.18) | 0.35 (0.13, 0.84) | 0.5 |
|  |  |  |  | Cattle | 53.81 (49.10, 58.45) | 0.28 (0.03, 0.84) | 0 |
|  |  |  |  | **Poultry** | **57.09 (51.02, 62.95)** | **1.47 (1.17, 1.62)** | **42.3** |
|  |  |  |  | Dog | 38.46 (31.7, 45.7) | 0.28 (0.05, 0.84) | 0 |
|  |  |  |  | Pig | 0.70 (0.24, 2.05) | 0.28 (0.02, 0.84) | 0 |
| 3 (7) | Penglai Prefecture, Shandong Province | 2011 | 1.25 (1.07, 1.34) | Goat/sheep | 69.66 (63.90, 74.87) | 0.35 (0.11, 0.87) | 0.6 |
|  |  |  |  | Cattle | 67.48 (62.80, 71.84) | 0.30 (0.05, 0.87) | 0 |
|  |  |  |  | **Poultry** | **37.97 (32.35, 43.93)** | **1.23 (1.07, 1.33)** | **41.5** |
|  |  |  |  | Dog | 37.29 (30.51, 44.61) | 0.30 (0.06, 0.87) | 0.1 |
|  |  |  |  | Pig | 5.57 (3.74, 8.22) | 0.30 (0.03, 0.87) | 0 |
| 4 (33) | Laizhou Prefecture, Shandong Province | 2011 | 1.41 (1.17, 1.61) | Goat/sheep | 74.77 (65.96, 81.93) | 0.39 (0.16, 0.83) | 1 |
|  |  |  |  | Cattle | 57.07 (49.84, 64.00) | 0.28 (0.03, 0.82) | 0 |
|  |  |  |  | **Poultry** | **52.14 (43.16, 60.98)** | **1.39 (1.16, 1.60)** | **40.3** |
|  |  |  |  | Dog | 35.85 (24.30, 49.31) | 0.29 (0.05, 0.82) | 0.1 |
|  |  |  |  | Pig | 0.00 (0.00, 2.14) | 0.28 (0.01, 0.82) | 0 |
| 5 (34) | Wuxi Prefecture, Jiangsu Province | 2010-2011 | 1.02 (1.01, 1.07) | Goat/sheep | 6.76 (2.92, 14.86) | 0.25 (0.06, 0.86) | 0.1 |
|  |  |  |  | Poultry | 1.27 (0.35, 4.53) | 0.62 (0.23, 0.97) | 5.5 |
|  |  |  |  | Dog | 0.00 (0.00, 20.39) | 0.27 (0.04, 0.86) | 0.3 |
|  |  |  |  | Pig | 0.00 (0.00, 4.05) | 0.30 (0.07, 0.86) | 0.6 |
|  |  |  |  | **Rodent** | **2.13 (0.59, 7.43)** | **0.80 (0.33, 1.00)** | **11.3** |
|  |  |  |  | Hedgehog | 0.00 (0.00, 22.81) | 0.52 (0.11, 0.96) | 5.8 |
| 6 (35) | Shangcheng and Guangshan Counties of Henan Province | 2012 | 2.53 (1.42, 4.20) | Goat/sheep | 76.27 (64.03, 85.31) | 1.13 (0.80, 1.91) | 5.2 |
|  |  |  |  | **Cattle** | **100.00 (94.25, 100.00)** | **2.32 (1.29, 4.03)** | **31.1** |
|  |  |  |  | Dog | 75.00 (59.81, 85.81) | 0.81 (0.36, 1.06) | 0.7 |
|  |  |  |  | Pig | 3.57 (0.98, 12.12) | 0.71 (0.09, 0.95) | 0 |
|  |  |  |  | Rodent | 1.06 (0.05, 5.78) | 0.71 (0.12, 0.95) | 0 |
| 7 (36) | Yixing, Liyang, Lishui, Jiangning, Xuyi, and Donghai Counties of Jiangsu Province | 2012-2013 | 1.29 (1.03, 1.61) | **Goat/sheep** | **66.79 (61.04, 72.07)** | **1.20 (1.01, 1.58)** | **26** |
|  |  |  |  | Cattle | 28.18 (22.65, 34.46) | 0.60 (0.08, 0.97) | 0.1 |
|  |  |  |  | Poultry | 1.29 (0.65, 2.52) | 0.63 (0.14, 0.97) | 1.1 |
|  |  |  |  | Dog | 7.40 (4.98, 10.85) | 0.60 (0.07, 0.97) | 0 |
|  |  |  |  | Pig | 4.66 (2.93, 7.33) | 0.61 (0.12, 0.97) | 0.4 |
|  |  |  |  | Rodent | 4.36 (3.19, 5.92) | 0.74 (0.34, 0.99) | 3.7 |
|  |  |  |  | Hedgehog | 2.67 (0.73, 9.21) | 0.61 (0.14, 0.97) | 0.3 |
| 8 (37) | Yixing County, Jiangsu Province | 2010-2011 | 1.02 (1.01, 1.07) | Goat/sheep | 13.79 (5.50, 30.56) | 0.53 (0.16, 0.96) | 2.2 |
|  |  |  |  | **Poultry** | **2.47 (0.68, 8.56)** | **0.96 (0.65, 1.02)** | **23.9** |
|  |  |  |  | Dog | 0.00 (0.00, 20.39) | 0.40 (0.08, 0.91) | 1.4 |
|  |  |  |  | Pig | 0.00 (0.00, 4.09) | 0.38 (0.07, 0.88) | 1.1 |
| 9 (38) | Pingqiao district and Xin County of Henan Province | 2016-2018 | 1.34 (1.13, 1.62) | Goat/sheep | 69.74 (58.67, 78.91) | 0.70 (0.32, 1.10) | 5.7 |
|  |  |  |  | Cattle | 97.92 (89.10, 99.89) | 0.65 (0.29, 1.03) | 4.3 |
|  |  |  |  | **Poultry** | **24.60 (18.98, 31.24)** | **0.91 (0.71, 1.07)** | **13.2** |
|  |  |  |  | Dog | 68.18 (53.44, 80.00) | 0.46 (0.14, 0.87) | 1.1 |
|  |  |  |  | Pig | 3.17 (0.87, 10.86) | 0.38 (0.07, 0.83) | 0.1 |
|  |  |  |  | Rodent | 7.69 (3.03, 18.17) | 0.45 (0.15, 0.85) | 1.2 |
|  |  |  |  | Hedgehog | 40.00 (19.82, 64.25) | 0.69 (0.40, 0.92) | 5.9 |
|  |  |  |  | Weasel | 91.11 (79.27, 96.49) | 0.43 (0.10, 0.85) | 0.7 |
|  |  |  |  | Hare | 63.01 (51.55, 73.18) | 0.47 (0.16, 0.85) | 1 |
|  |  |  |  | Wild bird | 37.84 (24.06, 53.90) | 0.52 (0.19, 0.89) | 2.4 |
