# Supplementary material for: Contributions of different host species to the natural transmission of severe fever with thrombocytopenia syndrome virus in China
Source: PLoS Negl Trop Dis. 2025 Jul 17;19(7):e0013304. doi: 10.1371/journal.pntd.0013304 (PMC12286343; doi:10.1371/journal.pntd.0013304)
Supplement: S1 Fig — The dots represent the point estimates, while the error bars represent the 95% confidence interval determined with the Wilson score interval method. The sizes of the dots represent the total sample size aggregated across studies, while the colors of the dots represent the number of studies that surveyed each species. (DOCX) [file pntd.0013304.s005.docx]

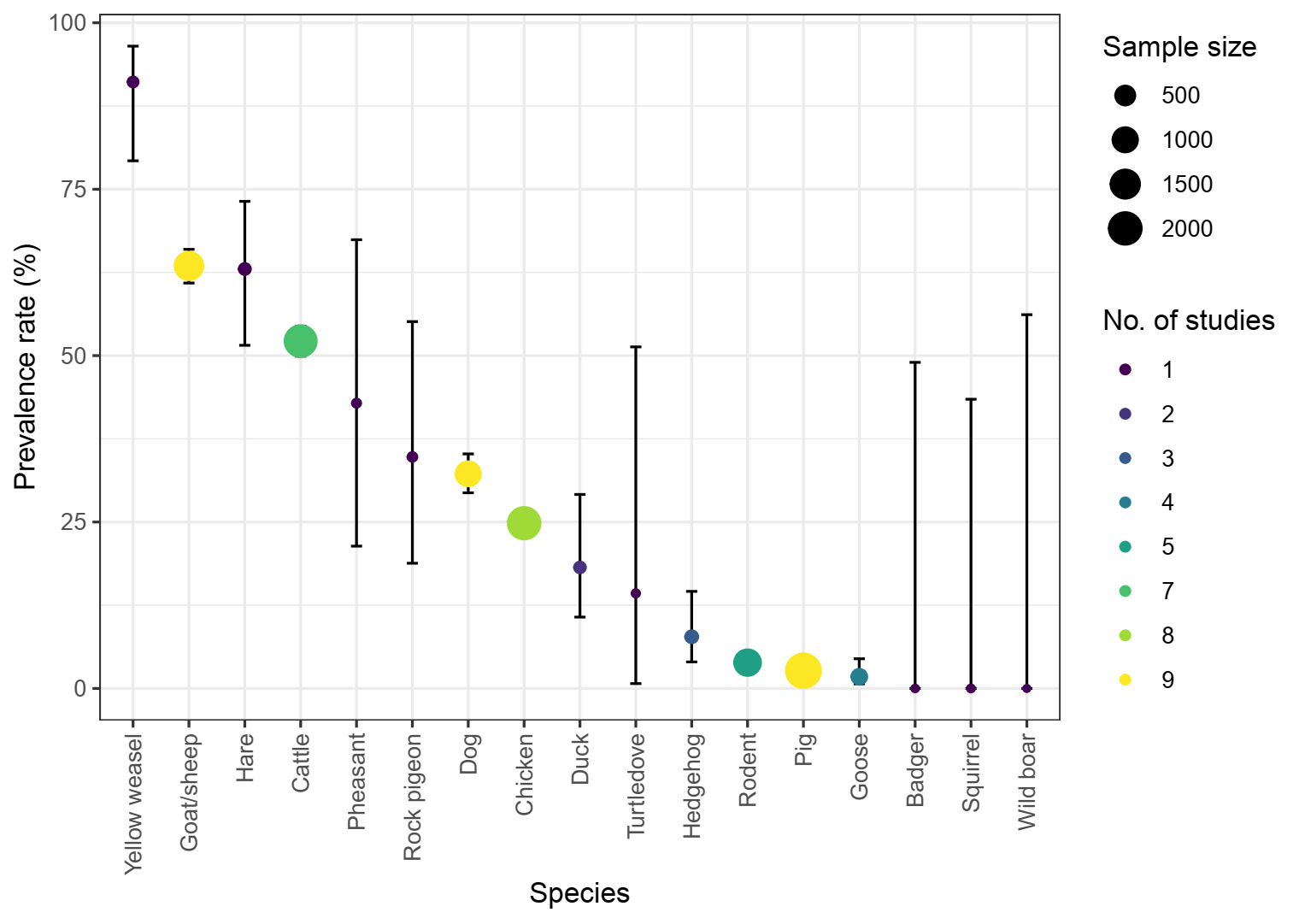


**Fig S1. Seroprevalence rates of SFTSV in different animal species.** The dots represent the point estimates, while the error bars represent the 95% confidence interval determined with the Wilson score interval method. The sizes of the dots represent the total sample size aggregated across studies, while the colors of the dots represent the number of studies that surveyed each species.
