# Supplementary material for: Contributions of different host species to the natural transmission of severe fever with thrombocytopenia syndrome virus in China
Source: PLoS Negl Trop Dis. 2025 Jul 17;19(7):e0013304. doi: 10.1371/journal.pntd.0013304 (PMC12286343; doi:10.1371/journal.pntd.0013304)
Supplement: S3 Fig — The length of the bar represents the median permutation importance score from 100 repetitions of fitting, each with 200,000 random rows (~one percent of the full dataset), while the error bar represents its 95% CI. (DOCX) [file pntd.0013304.s007.docx]

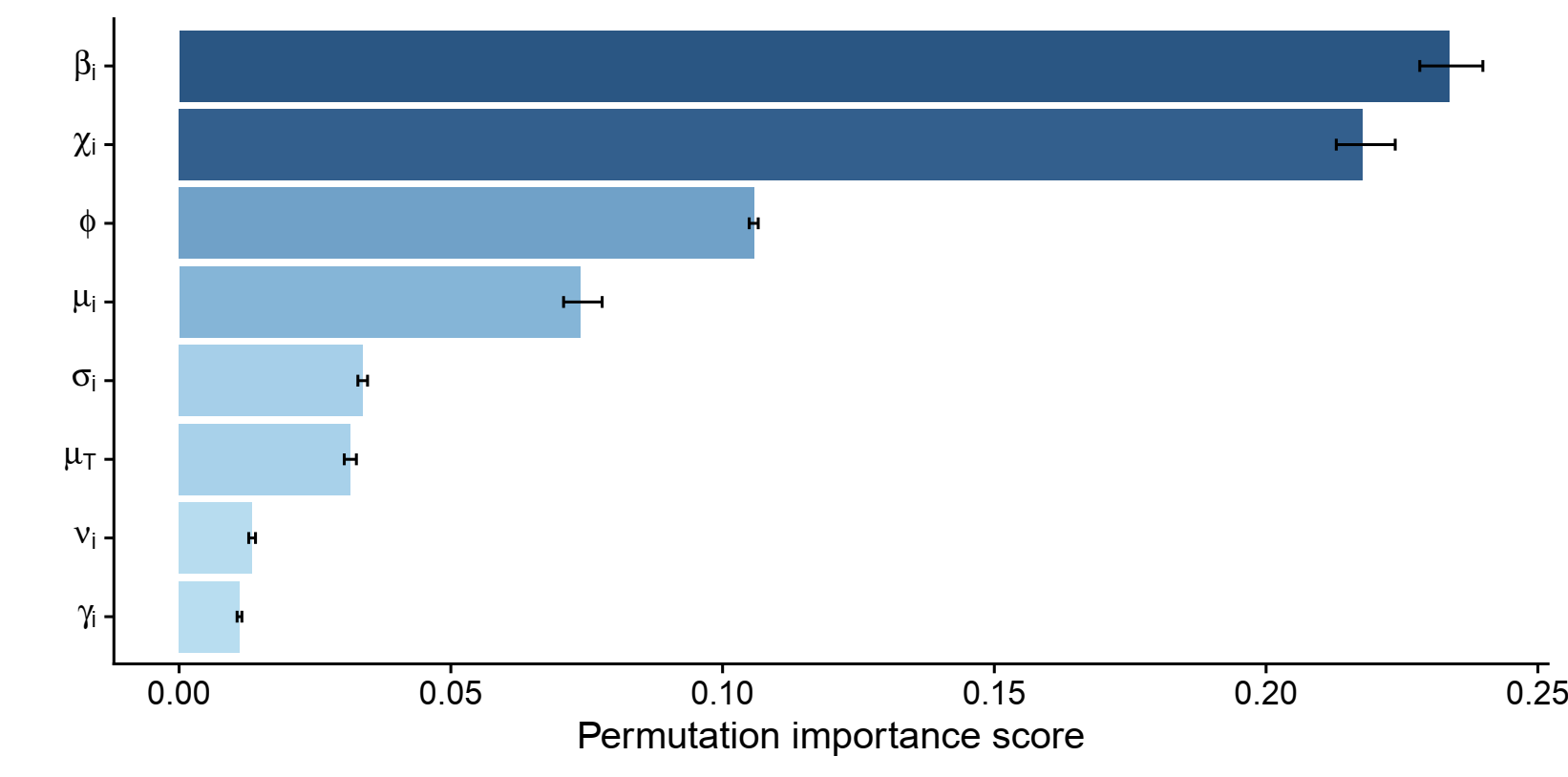


**Fig S3. Importance of each parameter in predicting the species-level** $\boldsymbol{R}_{\mathbf{0i}}$**.** The length of the bar represents the median permutation importance score from 100 repetitions of fitting, each with 200,000 random rows (~one percent of the full dataset), while the error bar represents its 95% CI.
