# Supplementary material for: Contributions of different host species to the natural transmission of severe fever with thrombocytopenia syndrome virus in China
Source: PLoS Negl Trop Dis. 2025 Jul 17;19(7):e0013304. doi: 10.1371/journal.pntd.0013304 (PMC12286343; doi:10.1371/journal.pntd.0013304)
Supplement: S4 Fig — Each dot represents the re-estimated R0i (y-axis) of a specific species (colors) after multiplying the parameter of interest (corresponding to the panel name) by a scaling factor (x-axis). The sizes of the dots represent the proportion of parameter sets that resulted in species-level seroprevalence rates within the confidence intervals of their observed values. When all parameter sets failed to match the seroprevalence rates, no dots were displayed. To assess the importance of each individual species’ abundance, χis, instead of χmax, were perturbed. The definition of each parameter can be found in S2 Text. (DOCX) [file pntd.0013304.s008.docx]

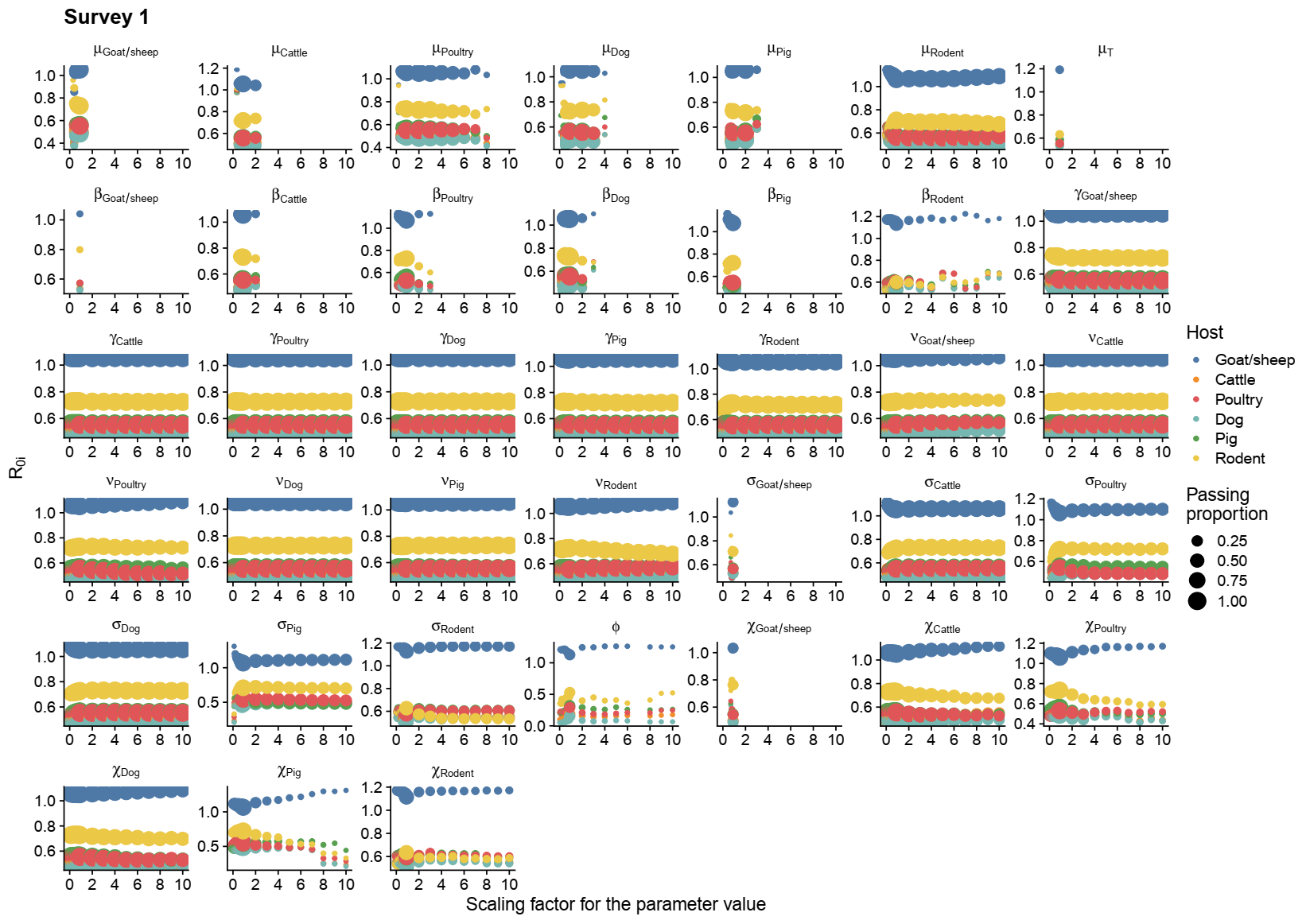

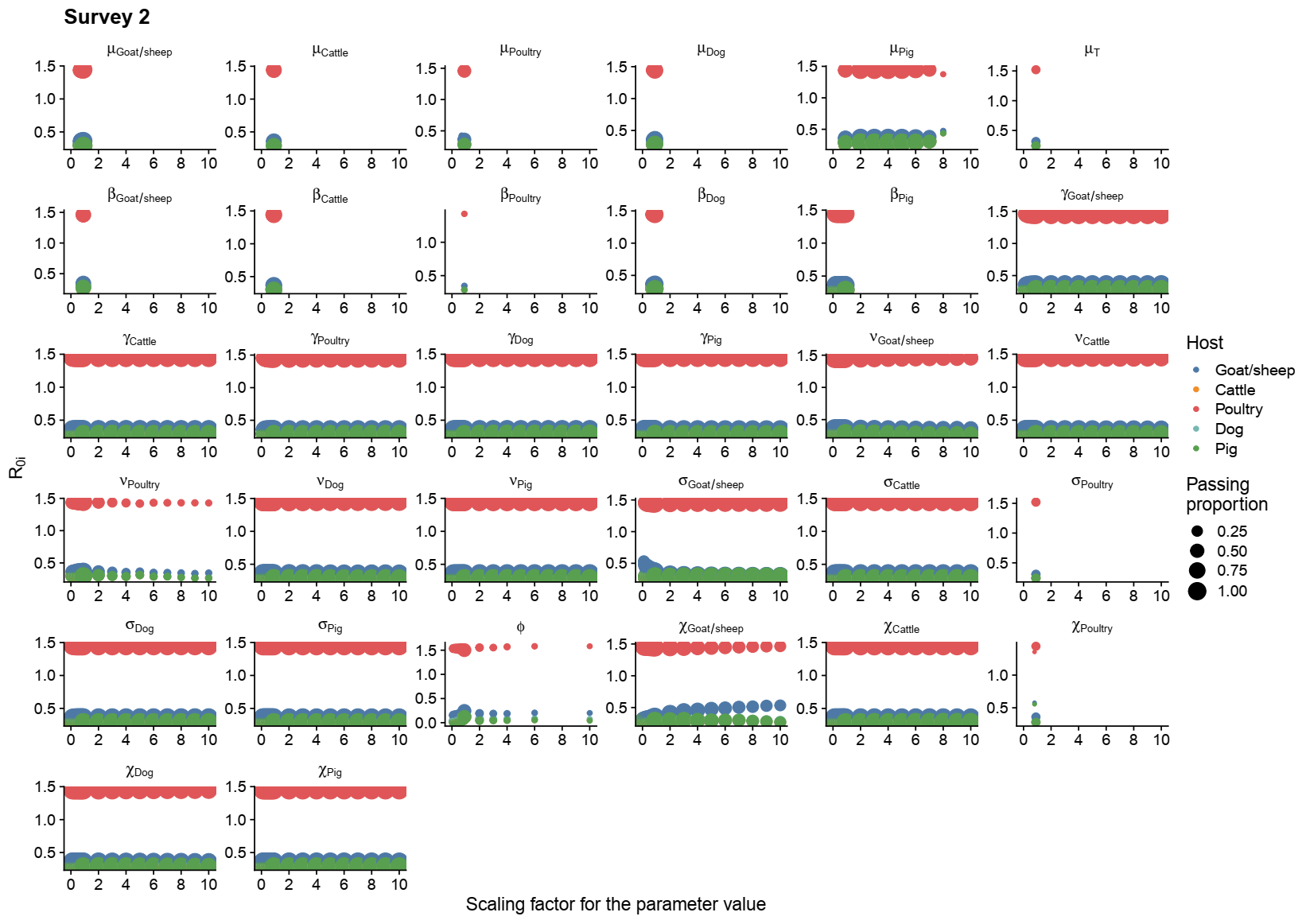

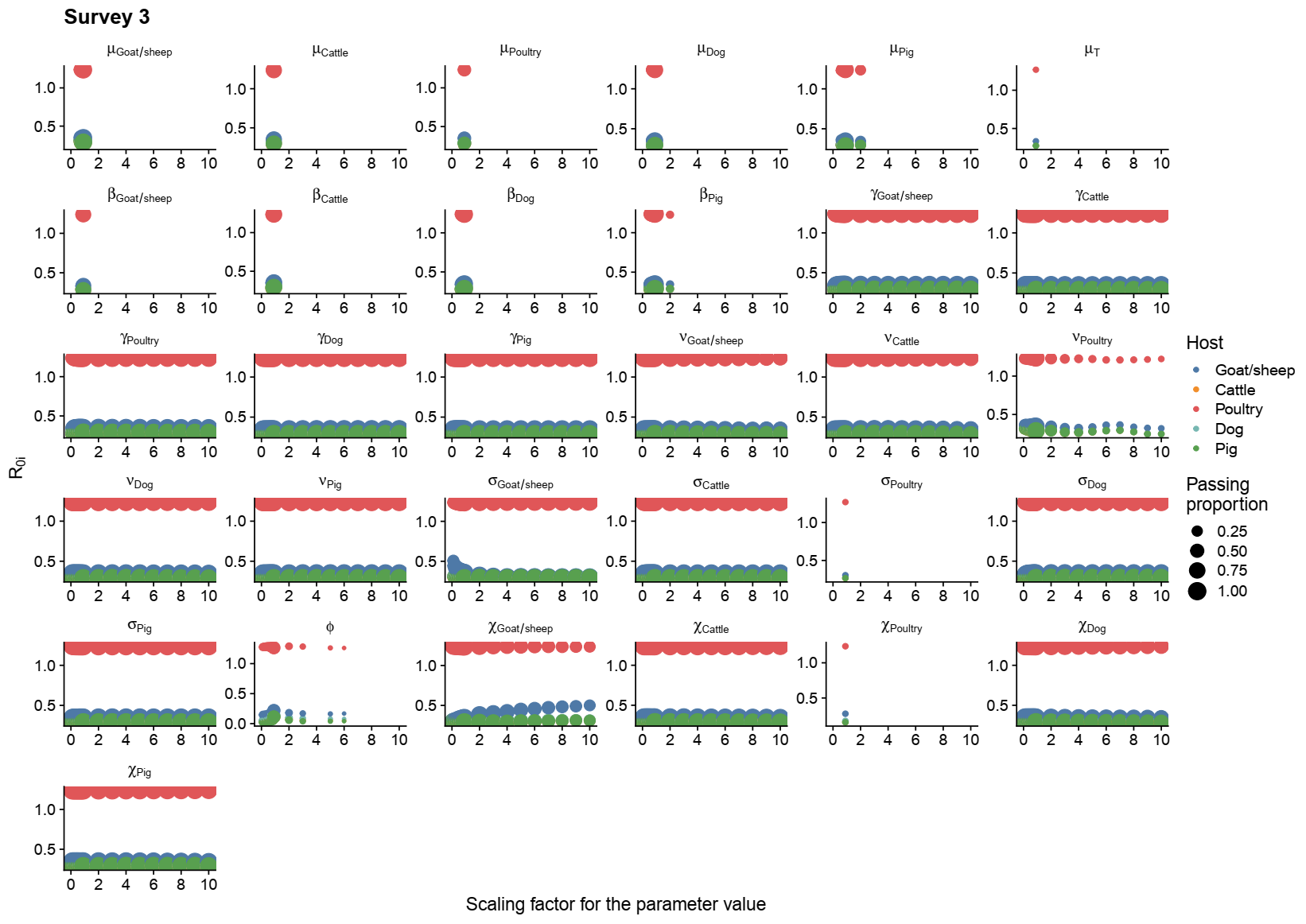

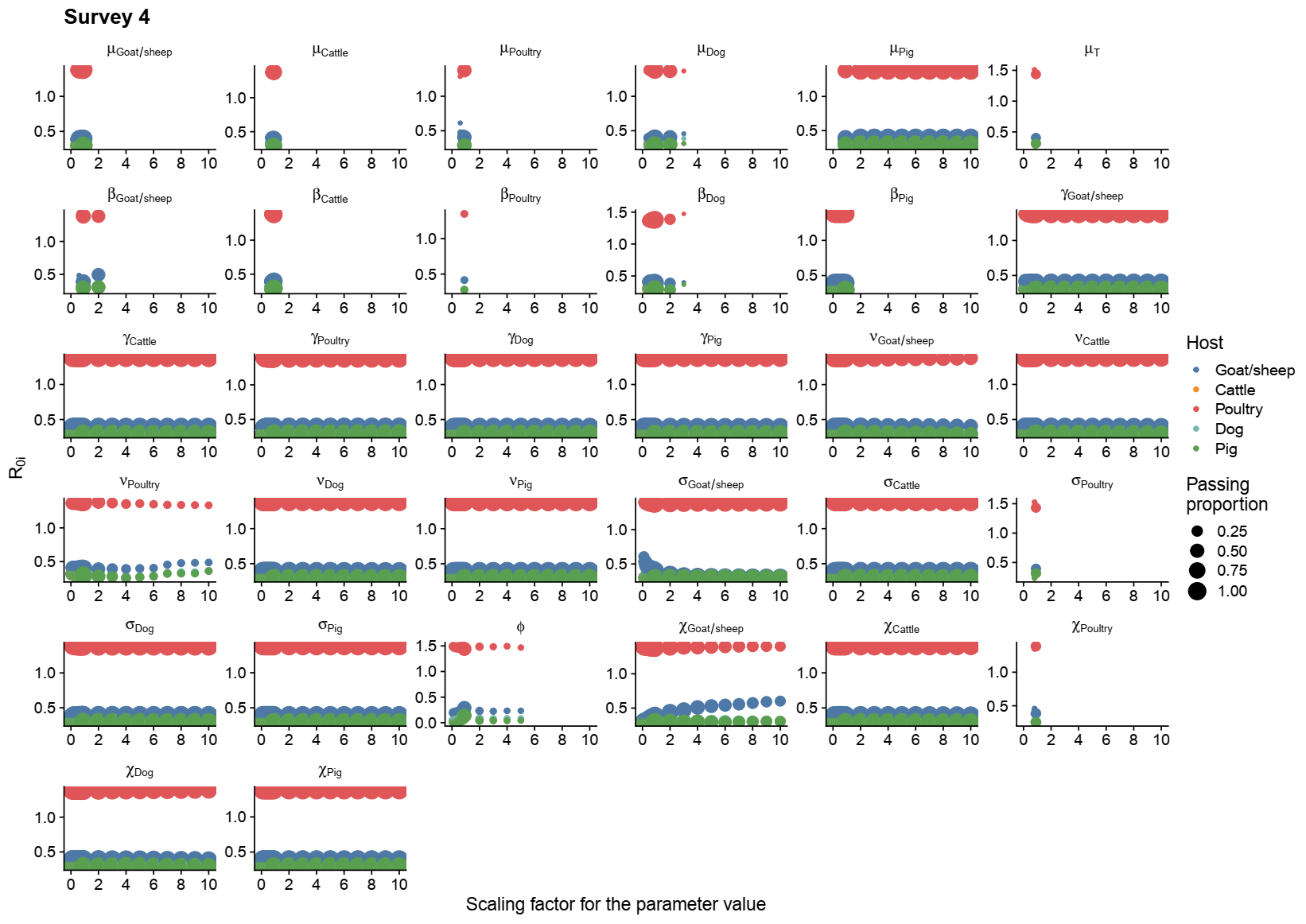

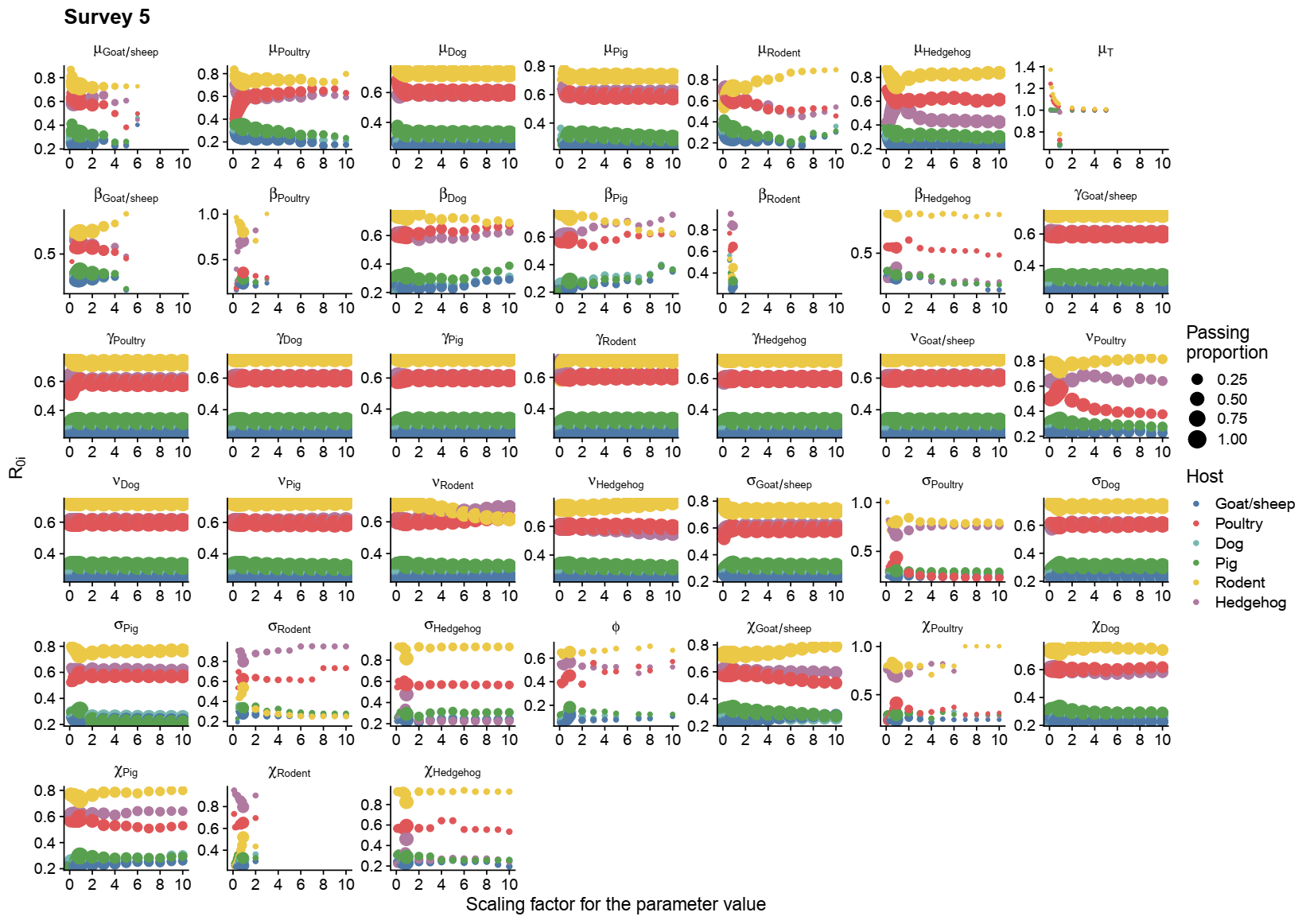

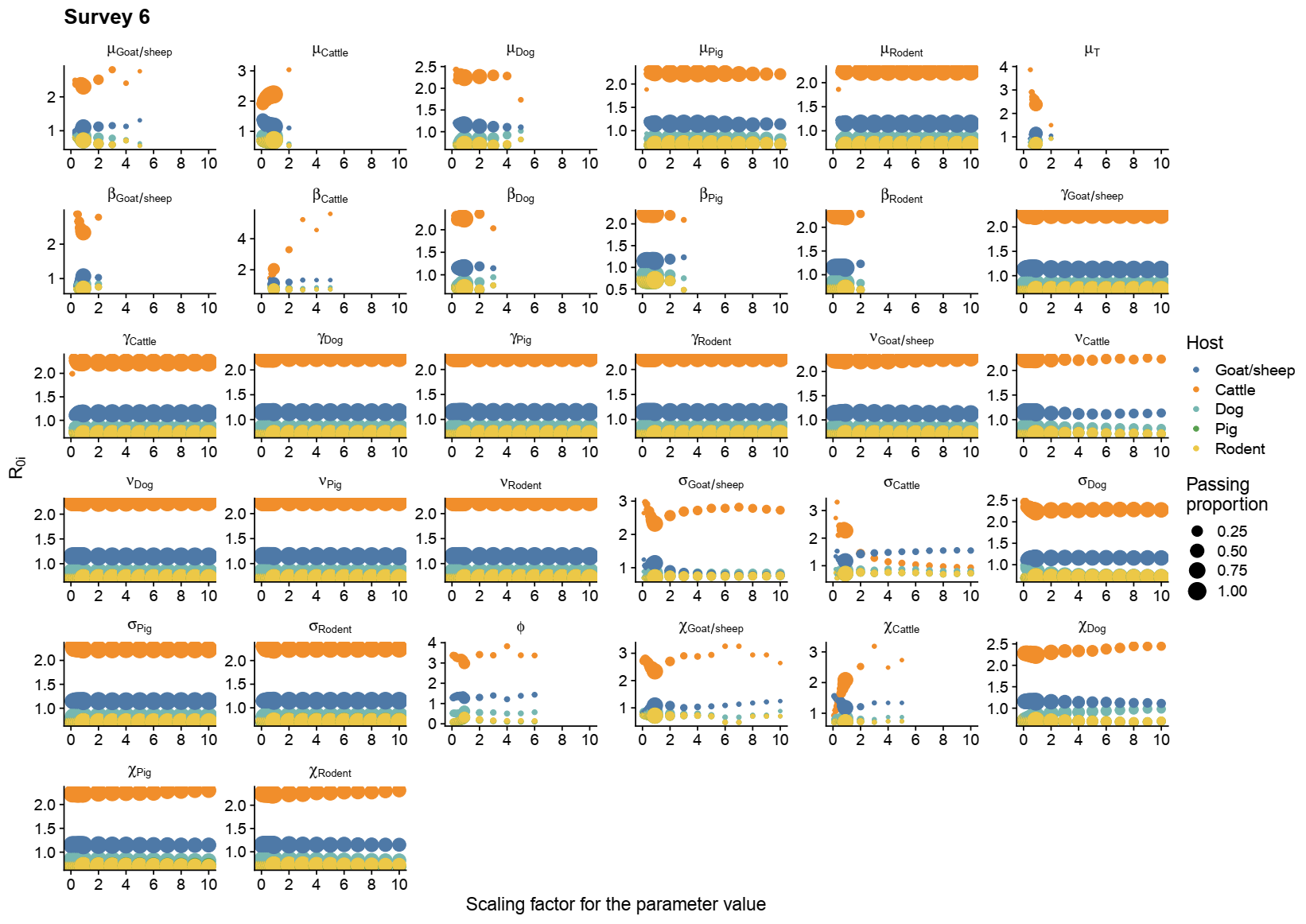

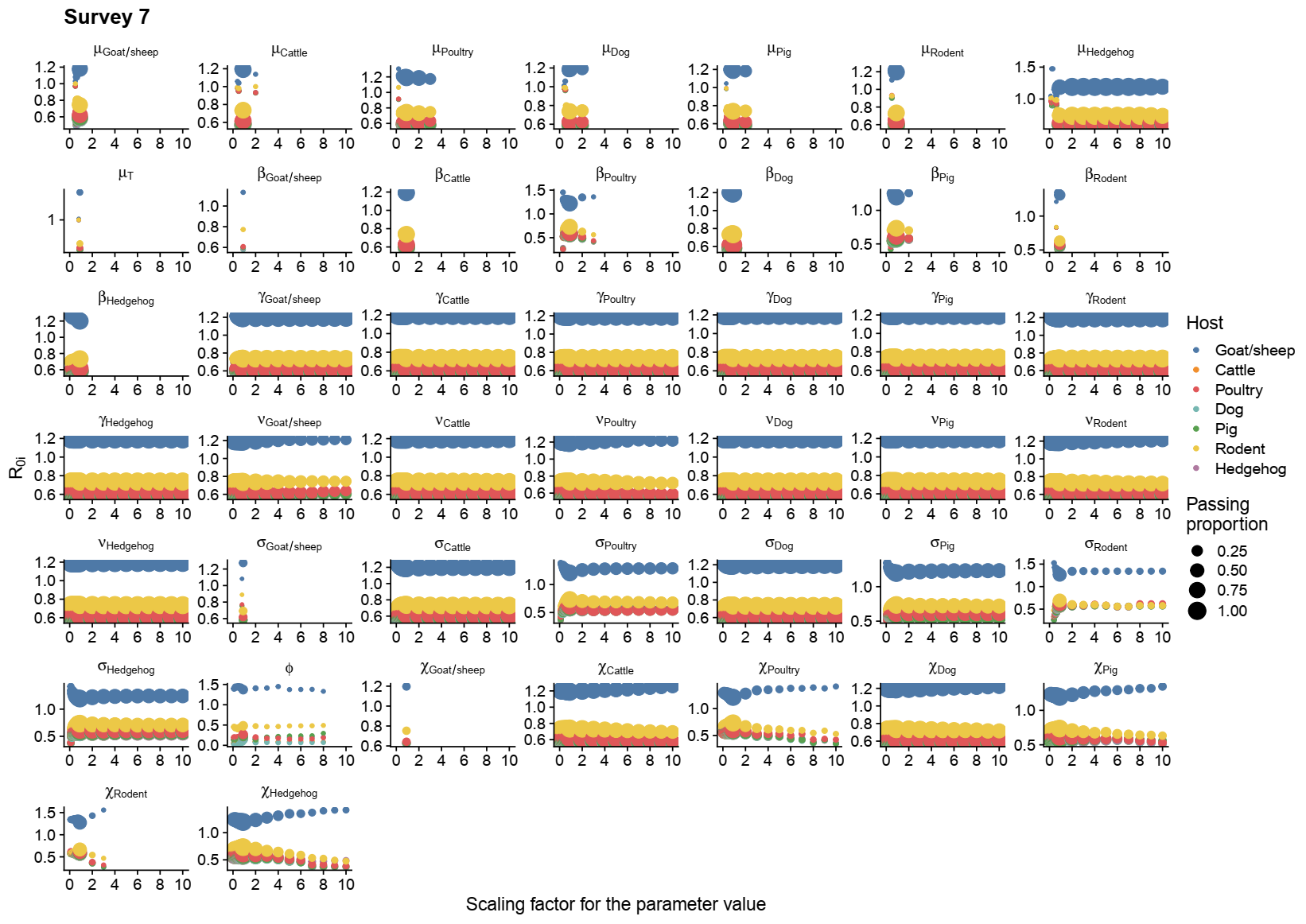

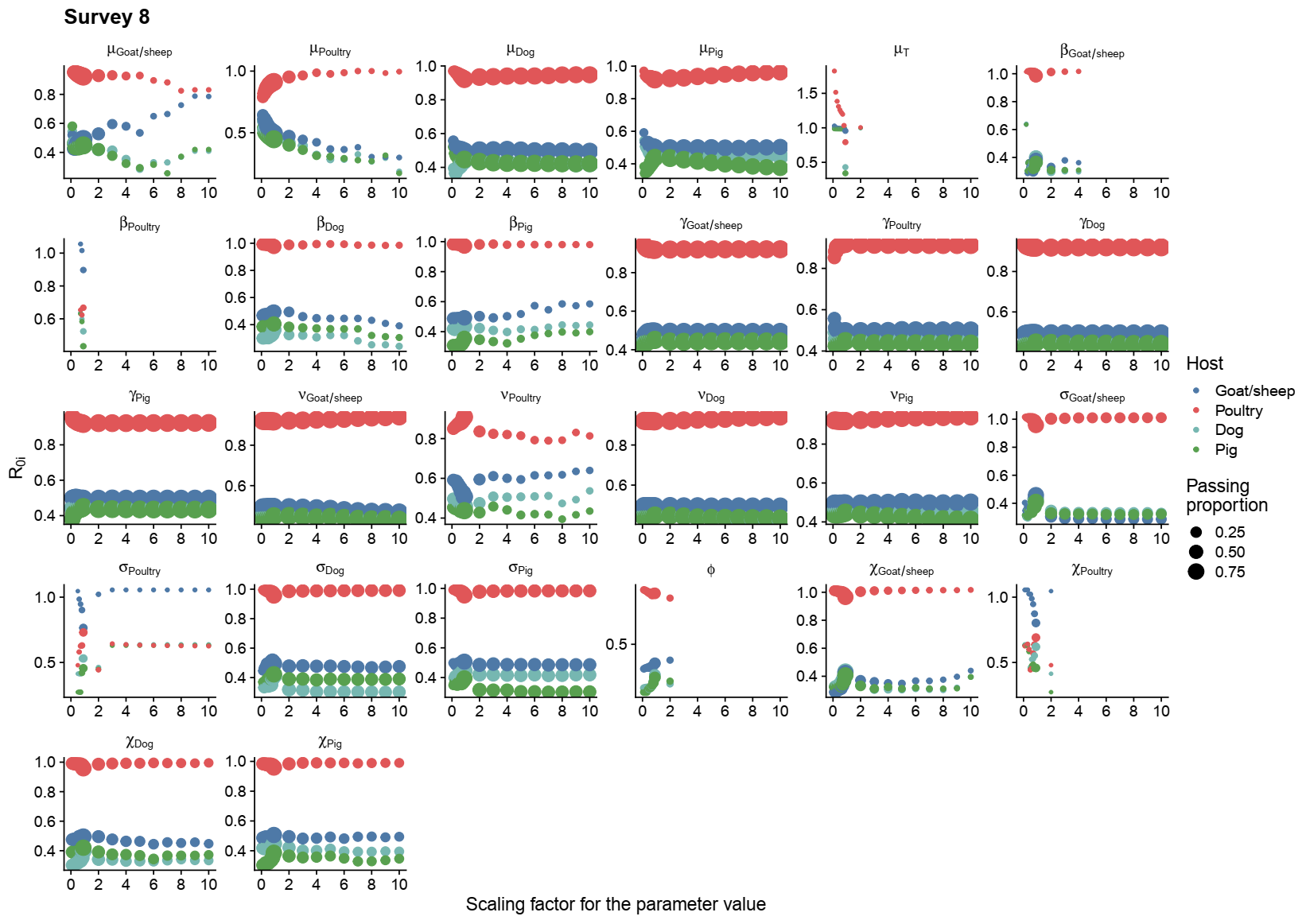

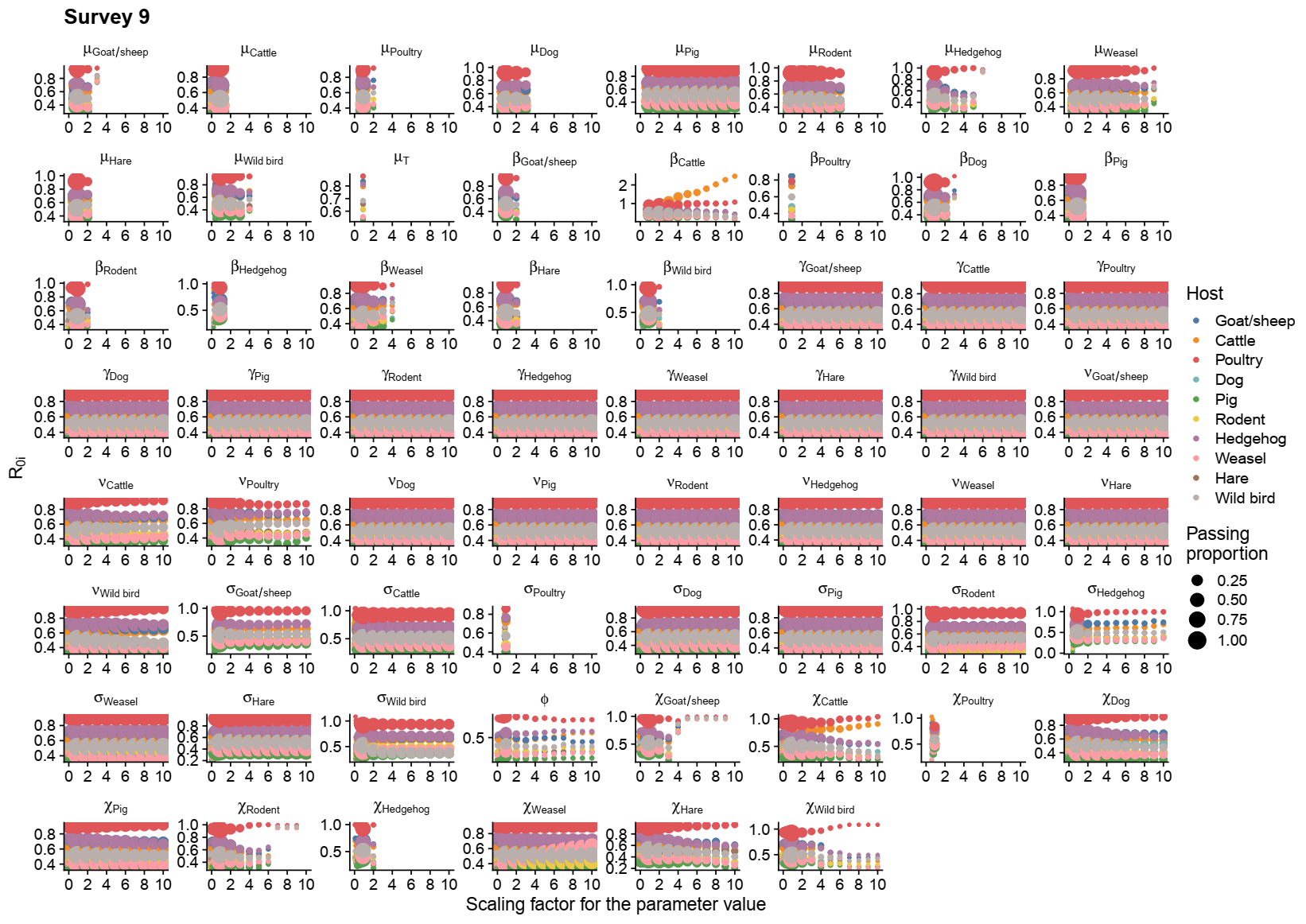


**Fig S4. Sensitivity of species-level** $\boldsymbol{R}_{\mathbf{0i}}$**s to changes of parameter values.** Each dot represents the re-estimated $R_{0i}$ (y-axis) of a specific species (colors) after multiplying the parameter of interest (corresponding to the panel name) by a scaling factor (x-axis). The sizes of the dots represent the proportion of parameter sets that resulted in species-level seroprevalence rates within the confidence intervals of their observed values. When all parameter sets failed to match the seroprevalence rates, no dots were displayed. To assess the importance of each individual species’ abundance, $\chi_{i}$s, instead of $\chi_{max}$, were perturbed. The definition of each parameter can be found in S2 Text.
