# Supplementary material for: Contributions of different host species to the natural transmission of severe fever with thrombocytopenia syndrome virus in China
Source: PLoS Negl Trop Dis. 2025 Jul 17;19(7):e0013304. doi: 10.1371/journal.pntd.0013304 (PMC12286343; doi:10.1371/journal.pntd.0013304)
Supplement: S5 Fig — X-axis represent the magnitude of the change, while y-axis represent the overall R0. (DOCX) [file pntd.0013304.s009.docx]

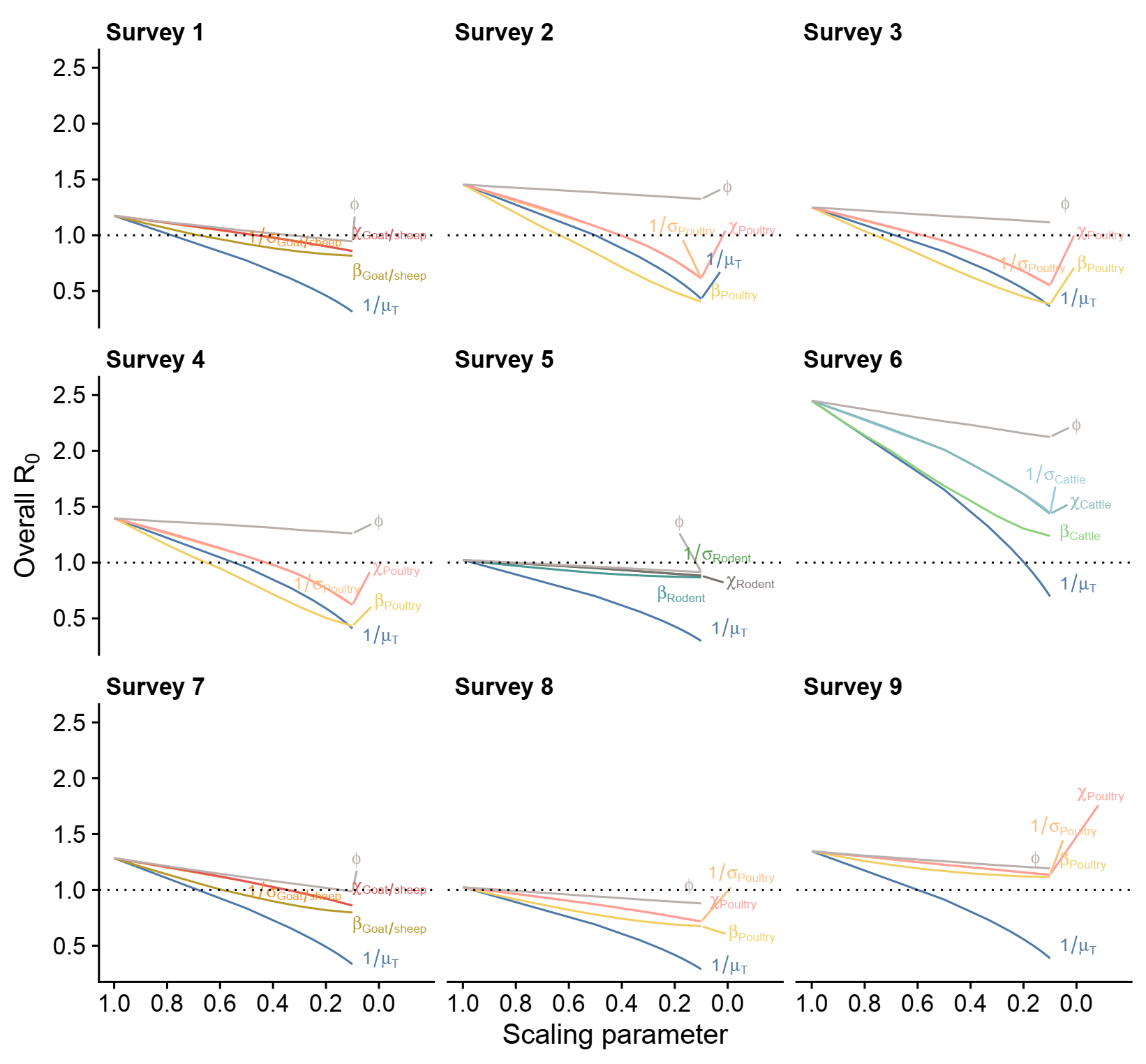


**Fig S5. The five interventions that can lead to the largest reduction in the overall** $\boldsymbol{R}_{\boldsymbol{0}}$ **for each location.** X-axis represent the magnitude of the change, while y-axis represent the overall $R_{0}$.
