# Supplementary material for: Contributions of different host species to the natural transmission of severe fever with thrombocytopenia syndrome virus in China
Source: PLoS Negl Trop Dis. 2025 Jul 17;19(7):e0013304. doi: 10.1371/journal.pntd.0013304 (PMC12286343; doi:10.1371/journal.pntd.0013304)
Supplement: S6 Fig — Black bars represent the values estimated using the full dataset, while the colored bars represent the values estimated when the seroprevalence rate for one host species was left out from the calibration. (DOCX) [file pntd.0013304.s010.docx]

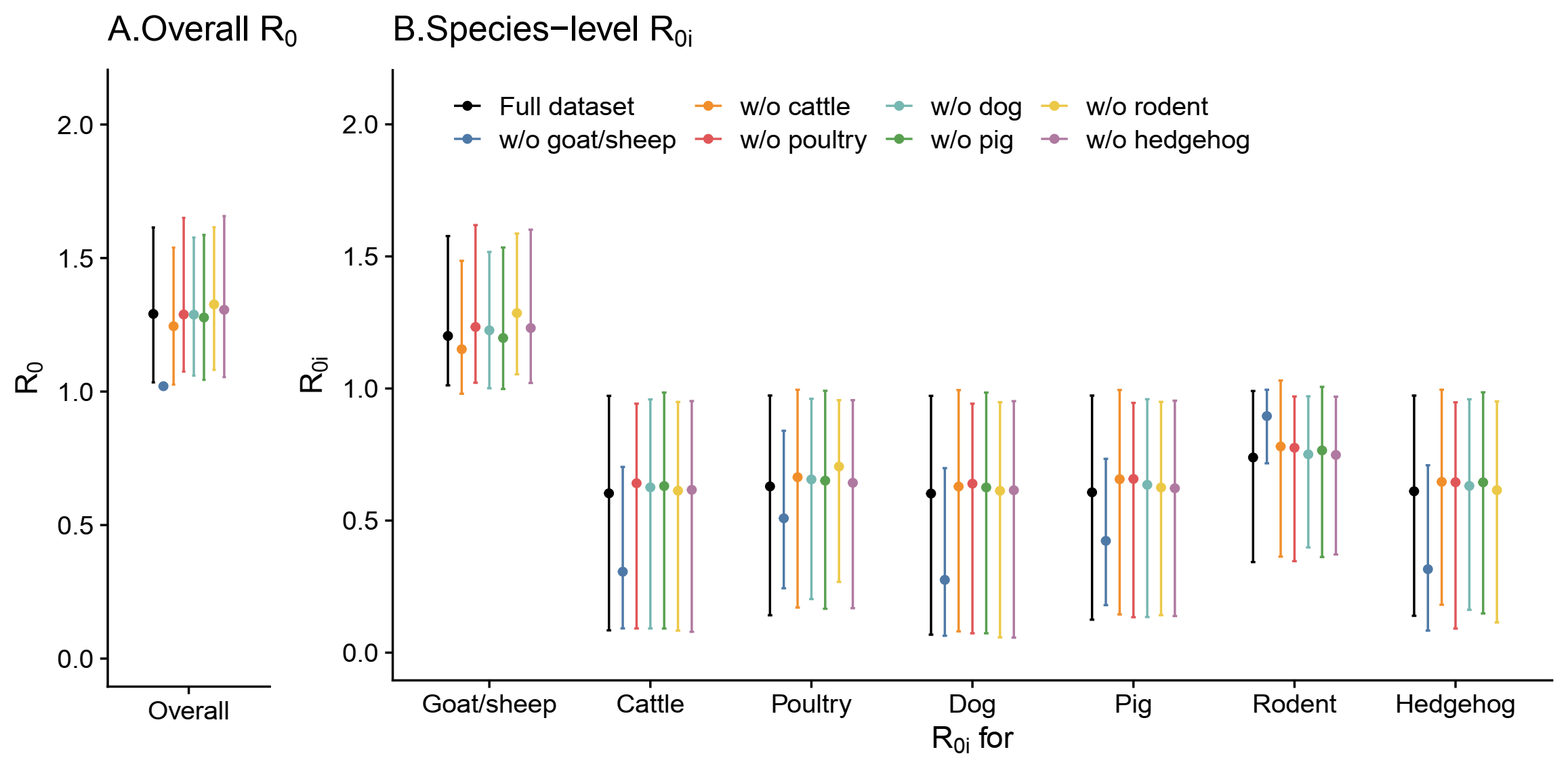


**Fig S6. (A) Overall** $\boldsymbol{R}_{\boldsymbol{0}}$ **and (B) species-level** $\boldsymbol{R}_{\boldsymbol{0}\boldsymbol{i}}$**s when different species was left out from the calibration for Survey 7.** Black bars represent the values estimated using the full dataset, while the colored bars represent the values estimated when the seroprevalence rate for one host species was left out from the calibration.
