# Supplementary material for: Contributions of different host species to the natural transmission of severe fever with thrombocytopenia syndrome virus in China
Source: PLoS Negl Trop Dis. 2025 Jul 17;19(7):e0013304. doi: 10.1371/journal.pntd.0013304 (PMC12286343; doi:10.1371/journal.pntd.0013304)
Supplement: Attachment — Submitted filename: Response letter.docx [file pntd.0013304.s012.docx]

| QU CHENG, PHD SCHOOL OF PUBLIC HEALTH  HUAZHONG UNIVERSITY OF SCIENCE AND TECHNOLOGY  WUHAN, CHINA 430030 | June 10, 2025 |
| --- | --- |

Dear Editors,

We are pleased to resubmit our research manuscript entitled “*Contributions of different host species to the natural transmission of severe fever with thrombocytopenia syndrome virus in China*” (Manuscript ID: PNTD-D-25-00557) for your consideration in PLoS Neglected Tropical Diseases*.*

We sincerely appreciate the insightful and constructive comments provided by the reviewers, which have helped strengthen the manuscript substantially. We agree with many of their suggestions and have revised the manuscript accordingly. Detailed responses to each comment are provided below.

We look forward to hearing from you regarding our revision, and we would be glad to respond to any further comments the editors or reviewers may have.

Sincerely,

Qu Cheng, Ph.D. [chengqu@hust.edu.cn](mailto:chengqu@hust.edu.cn)

Department of Epidemiology and Biostatistics, School of Public Health, Tongji Medical College

Huazhong University of Science and Technology

Reviewer #1: 1. Line 42: Huaiyangshan Banyangvirus was a provisional name previously used; however, the virus has since been officially named Dabie bandavirus by the International Committee on Taxonomy of Viruses (ICTV). Therefore, I recommend deleting the former name. Additionally, since the current name was established by the ICTV in 2020, it would be appropriate to include a reference to support this information.

**We appreciate the reviewer’s comment and agree that *Huaiyangshan banyangvirus* is an outdated name. However, we believe that retaining all previously used names may help avoid confusion for readers familiar with historical terminology. To address this, we have revised the sentence to clearly indicate that *Bandavirus dabieense* is the most recent ICTV-approved name, with former names included parenthetically for reference. A citation to ICTV has also been added. The revised sentence now reads:**

**L58 (track changes version):** “Severe fever with thrombocytopenia syndrome virus (SFTSV), also known as *Bandavirus dabieense* (formerly *Dabie bandavirus* and *Huaiyangshan banyangvirus*), is the causative agent of SFTS, an emerging tick-borne zoonotic disease.”

2. Line 46-48: The term dead-end hosts is currently not well-supported by sufficient evidence. References 12 and 13, which are cited in support of this term, are not directly relevant to dead-end host dynamics. This sentence should be restructured accordingly to avoid potentially misleading interpretations

**We agree. In response, we have revised the sentence to emphasize the limited role of humans in the natural persistence of SFTSV and removed the term “dead-end hosts”:**

**L64 (track changes version):** “Though human-to-human transmission of SFTSV can occur via several routes [13, 14], it is much less frequent than tick-human transmission [15], such that infected humans contribute minimally to the natural transmission of SFTSV.”

3. The analysis in the manuscript is based on seroprevalence data, primarily derived from ELISA-based studies. It should be noted that ELISA assays using SFTSV-NP as the antigen have been reported to cross-react with other bunyaviruses, as shown in previous Chinese studies. To ensure accurate information delivery to readers, a brief summary of the methodologies used to determine seropositivity should be included.

**We appreciate the reviewer’s insightful comment and have revised the manuscript to clarify the methodologies used to determine seropositivity in previous studies, as well as the potential for cross-reactivity at L122 of the track changes version:**

“All the included studies used a double-antigen sandwich enzyme-linked immunosorbent assay (ELISA) targeting the nucleocapsid protein of SFTSV to detect total antibodies, which may also cross-react with other less common bandaviruses, such as Guertu virus and Heartland virus.”

4. In the section discussing the limitations of the study, further consideration should be given to the interpretation of SFTSV-specific total IgG levels. One possible explanation for the high antibody prevalence observed in animals could be interspecies differences in antibody half-life. Additionally, repeated exposure to SFTSV may result in a boosting effect, maintaining elevated antibody titers over time.

**We thank the reviewer for this suggestion and have expanded the limitations to explicitly address the interspecies variability in antibody dynamics:**

**L426 (track changes version):** “We also assumed lifelong antibody persistence across host species based on observations of long-lasting antibodies in humans; however, antibody half-lives remained unmeasured in non-human host species. Furthermore, repeated exposure to SFTSV in some host species may also sustain elevated antibody titers through immune boosting. These interspecies differences in antibody kinetics and exposure history may also contribute to the large variation in seroprevalence rates observed across host species. ”

5. The manuscript suggests that poultry may play a major role in the transmission of SFTSV. To support this claim, it would be more convincing to include a summary and discussion of research findings related to SFTSV RNA detection rates in poultry, clinical symptoms, and experimental infection studies. Since the mathematical model was based solely on existing seroprevalence data from China, it would be helpful to reference empirical studies that correlate with the modeling results derived from this "dry work."

**We fully agree with the reviewer that additional details related to SFTSV RNA detection rates, clinical symptoms, and experimental infection studies in poultry could further strengthen the discussion. However, as we mentioned in the original abstract and discussion, the potential role of poultry in SFTSV transmission has been largely overlooked. Although several studies have examined the RNA detection rate in poultry, the positivity rate is generally very low due to the short duration of viremia. Among the nine locations included in our study, RNA positivity rates were only reported at three locations for poultry, which ranged from 0 to 2.7%. Similarly, low rates were observed for other species as well, ranging from 0 to 6.6%, which may also be attributed to the short duration of viremia. Besides these locations included in our study, a cross-sectional survey from the Republic of Korea also reported a 2.5% RNA positivity rate in poultry. We have added a sentence in the revised manuscript to discuss the RNA positivity rate for poultry:**

**L130 (track changes version):** “Seroprevalence rates, rather than SFTSV RNA positivity rates, were used to calibrate the models because they were more frequently reported in the literature (i.e., only 3 out of 9 locations included in our study reported SFTSV RNA positivity rates) and were typically estimated with greater precision, due to their higher values (i.e., seroprevalence rates in poultry ranged from 0.84 to 57.09% in eligible studies, compared to 0 to 2.7% for RNA positivity rates) and consequently narrower confidence intervals.”

**As also mentioned in our original discussion, no studies to date have reported clinical symptoms or experimental infection results in poultry. The only relevant experimental study involves spotted doves (Li et al., 2009). In that study, spotted doves exhibited clinical symptoms like anorexia, weight loss, lethargy, and ruffled feathers following SFTSV challenge. The severity of the infection was dose-dependent: 62.5% of the birds developed viremia when challenged with 10^3^ PFU, whereas 100% developed viremia and 12.5% died when challenged with 10^7^ PFU. We have added a sentence to the Discussion section to recommend future research examining the susceptibility and clinical symptoms of SFTSV infection in poultry:**

**L424 (track changes version):** “Future experimental infection studies are needed in poultry to better understand their clinical symptoms, duration of viremia, case-fatality rate, and antibody dynamics.”

______________________________________________________________________

Reviewer #2: This manuscript presents exploration of a multihost mathematical model aimed at evaluating the role different tick host species play in the transmission of SFTS. The work combines species-specific seroprevalence estimates from across a number of reported surveys, with a simple but appropriate mathematical model, and a sensible approach to calibration, to identify the key hosts the drive transmission (contribute to total R0) in each survey, and implications for different targeted control strategies.

Overall the work is well presented, logical, and leads to some interesting insights – in particular highlighting the potential important role that poultry play in dominating transmission in many of the sites, but also showing how identity of the key host species can vary between locations. It also serves as a neat illustration of how limited data and simple mathematical models can be combined to reveal important insights, and generate new hypotheses about the roles different host species play in such complex systems. As such I think this paper has the potential to make a good contribution to the wider field. I do though have several points that I would like to see the authors address, primarily related to improving clarity in presentation and interpretation:

1.1 Line 147 – the simulated seroprevalence is given as the equilibrium ‘recovered’ (Ri) size of each host species. Potentially though, actively infected hosts (the Ii classes) would also test seropositive. I understand those may be quite short durations before recovery, but it may alter results if simulated seroprevalence is given as Ii+Ri. Have the authors assessed sensitivity of their results to this?

**Thank you for raising this important point. We agree that, in theory, actively infected hosts (the Ii compartments) could also contribute to seroprevalence if testing detects recent infections. However, given the very short infectious period, the proportion of individuals in the Ii compartments should be negligible. For instance, in our model, the average proportion of Ii individuals ranged from 0.0075% (pig, Survey 2) to 0.5% (hedgehog, Survey 9) across all species and surveys. To further address this concern, we conducted a sensitivity analysis using Ii + Ri to match the observed seroprevalence rates, instead of Ri alone. We used Survey 9 as an example, since it had the highest species-specific average proportion of individuals in the Ii compartment. As expected, the resulting estimates of both overall and species-specific R_0_ remained consistent with our original estimates, as shown in the table below. Given its minimal impacts on the results and to maintain focus on the core analysis, we opted not to include these results in the revised manuscript but would be happy to provide them as supplementary materials if the reviewers or editors think it would be helpful.**

|  | **Using Ri** | **Using Ii + Ri** |
| --- | --- | --- |
| **Overall R_0_** | 1.34 (1.13, 1.62) | 1.34 (1.21, 1.56) |
| **Species-specific R0** |  |  |
| Goat/sheep | 0.70 (0.32, 1.10) | 0.66 (0.29, 1.11) |
| Cattle | 0.65 (0.29, 1.03) | 0.61 (0.33, 0.98) |
| Poultry | 0.91 (0.71, 1.07) | 0.89 (0.72, 1.08) |
| Dog | 0.46 (0.14, 0.87) | 0.41 (0.16, 0.85) |
| Pig | 0.38 (0.07, 0.83) | 0.31 (0.06, 0.85) |
| Rodent | 0.45 (0.15, 0.85) | 0.42 (0.21, 0.85) |
| Hedgehog | 0.69 (0.40, 0.92) | 0.65 (0.38, 0.9) |
| Weasel | 0.43 (0.10, 0.85) | 0.37 (0.11, 0.85) |
| Hare | 0.47 (0.16, 0.85) | 0.42 (0.2, 0.86) |
| Wild bird | 0.52 (0.19, 0.89) | 0.52 (0.31, 0.9) |

1.2 More generally, it would be useful to have a little more information about the calibration process here. I found myself wondering how closely the simulated values had to ‘match’ the observed (I later saw in the Supp Info that they just had to fall within the 95% CIs of the observed prevalence values). Also it wasn’t immediately clear how parameter values were generated – again it is stated in the Supp Info that these were drawn randomly etc – but a little more detail in the main text will help the reader understand exactly what was done.

**Thank you for this helpful suggestion. We agree that providing additional details in the main text will improve readers’ understanding of the calibration process. To address this, we have revised the manuscript to describe how parameter values were sampled and how passes were defined. To maintain readability, we kept the description in the main text concise, while providing more technical detains in the Supplementary Text to avoid overwhelming readers.**

**L175 (tracked changes version):** “In brief, we randomly sampled parameters from the ranges defined in Table 1 until we obtained 1,000 sets of parameters that generated simulated seroprevalence rates (equilibrium values of $R_{i}$s) within the 95% confidence intervals of their observed values for all surveyed species (referred to as *passes*) and resampled them using the sampling-importance resampling approach [31, 32] to generate the posterior samples for estimating the overall and species-specific basic reproduction numbers.”

2. Line 178 – it isn’t clear why acaricide usage would reduce contact rates, instead of (or in addition to) increasing tick mortality. Is the assumption that acaricides provide a repellent protective effect to treated hosts?

**The application of acaricides was indeed assumed to have dual effects of both reducing the contact rate between ticks and hosts and increasing tick mortality, as described in the original sentence “***…, such as the application of acaricides to reduce the contact rate between host species and ticks (*$\beta_{i}s$*) and to increase tick mortality (increase* $\mu_{T}$*), …***”. We did assume that acaricides can have a repellent effect according to a few previous studies (Hagimori et al., 2005; Duscher et al., 2013). However, we understand that the killing effect of acaricides is more widely accepted than its repellent effects. To avoid confusion, we have revised the sentence to use essential oils, which are more commonly associated with repellent effects, as the example intervention for reducing contact rates:**

**L207 (track changes version):** “Various interventions can be implemented to mitigate the spread of SFTSV, such as the use of essential oils to reduce the contact rate between host species and ticks ($\beta_{i}s$) due to repellent effects, the application of acaricides to increase tick mortality (increase $\mu_{T}$), culling to decrease the abundance of host species ($\chi_{i}s$), treatment of diseased hosts to shorten their infectious periods (1/$\sigma_{i}s$), and the development of genetic modification techniques to reduce the efficiency of transovarial transmission of SFTSV ($\phi$) in ticks.”

3. Figure 2 – panel B gives a nice visual representation of species-specific prevalences, but these in themselves are of limited value as indicators of contributions to transmission, as host abundance is very important (as shown in the expressions for the R0i, and subsequent results) – it is the number of infecteds that drive transmission. Could equivalent plots of Figure 2B be provided that show estimated host abundance across the different surveys, or even calculated numbers infected (seroprevalence x host abundance)?

**We sincerely appreciate this insightful suggestion. In response, we have added two new panels to the revised Figure 2. Panel C represents the estimated abundance of each host species across different surveyed prefectures, and panel D represents the estimated number of seropositive individuals for each species (calculated as seroprevalence * host abundance). We fully agree that adding these panels can help provide a more comprehensive view of the potential contribution of each host species to the natural persistence of SFTSV, and notably highlight the possible significant role of poultry in the transmission dynamics. We have also added the following sentences to describe the new panels at L270 of the track changes version:**

“As shown by the expressions for $R_{0}$ and $R_{0i}$, the abundance of each host species critically influences local transmission intensity. While poultry and rodents exhibit the largest estimated population size (Text S2) across surveyed locations (Figure 2C), poultry contributed disproportionately more seropositive individuals due to their remarkably higher seroprevalence rates compared to rodents (Figure 2D).”

4. Figure 3 gives a nice representation of the estimated relationships between Overall R0 and species-specific seroprevalences – however those are then used to infer that goats/sheep, cattle, poultry, dogs, hedgehogs “play a [more] important role…in sustaining transmission” (lines 244-245). But I think it’s very hard to infer cause-and-effect from relationships like this. It’s not impossible (confirmed by the later analyses) that for at least some of these species, they may act as ‘sentinels’, serving as an indicator of high transmission (ie, they show high seroprevalence when R0 is high) – but don’t necessarily play much of a role in driving high R0. Some care or extra nuance in interpreting these relationships may be useful.

**Thank you for raising this important point regarding the interpretation of correlation versus causation. We fully agree that the sentence should emphasize the potential use of these species as sentinels rather than implying that they played important roles to local transmission. To clarify this, we have revised the text as follows:**

**L286 (track changes version):** “This pattern implies that the seroprevalence rates of the former group were more strongly correlated with spatial variation in transmission intensity compared to the latter group, and can serve as sentinels for identifying regions with elevated SFTSV transmission intensity.”

5.1 ‘Impact of missing key host species’ – I understand the use of Survey 9 to explore the consequences of missing certain species, given it was the one with the greatest number surveyed – but any results from that are going to depend on the specific findings from that original survey. If, for example, Survey 7 was used (which had the next highest number of host species surveyed, and found that goats/sheep were the dominant transmission hosts) we would presumably see quite different outcomes. I think it’s nice for the authors to have carried out this assessment, but it should be made clear that the specific results depend strongly on the initial findings relating to Survey 9 specifically.

**We appreciate this constructive comment and agree that the findings could be influenced by specific characteristics of the survey used. To address this, we repeated the analysis using data from Survey 7, whose results did differ in some aspects from those based on Survey 9. Specifically, when goat/sheep, the species with both the highest relative contribution and the highest seroprevalence rate (66.79%) was excluded, the estimated R_0_ and R_0i_s decreased substantially, instead of increased remarkably as in Survey 9. This contrast likely reflect the differences in the distribution of species-specific seroprevalence and transmission contributions across the two surveys. In Survey 7, excluding the dominant species goat/sheep left only species with low seroprevalence rates (<28.18%), indicating lower transmission intensity, while in Survey 9, the remaining species after excluding poultry still had high seroprevalence rates (e.g., 69.74% for goat/sheep and 97.92% for cattle), suggesting a need to maintain high transmission intensity and redistributing contributions to the remaining species and ticks to compensate for the omitted species. Despite this difference, the analyses revealed high consistency as well – in both surveys, the estimated R_0_ and R_0i_s remained relatively stable unless the most important species was excluded. We have added a supplementary figure (Figure S6), and revised the main text to incorporate the results when excluding one species at a time from Survey 7:**

**Figure S6. (A) Overall** $\boldsymbol{R}_{\boldsymbol{0}}$ **and (B) species-level** $\boldsymbol{R}_{\boldsymbol{0}\boldsymbol{i}}$**s when different species was left out from the calibration for Survey 7.** Black bars represent the values estimated using the full dataset, while the colored bars represent the values estimated when the seroprevalence rate for one host species was left out from the calibration.

**L221 (track changes version):** “We used data from Surveys 9 and 7 as examples, since they had the largest and second-largest number of host species, respectively.”

**L337 (track changes version):** “When recalibrating the model to the seroprevalence rates of the remaining species after systematically omitting one species at a time from Surveys 9 or 7 (represented by bars with different colors, Figures 5 and S6), the overall $R_{0}$ (Figures 5A and S6A) and species-level $R_{0i}$ (Figures 5B and S6B) remained largely stable, except when the species with the highest $R_{0i}$ was left out. In Survey 9, excluding poultry, the species with highest relative contribution but relatively low seroprevalence rate, led to remarkable increase in both $R_{0}$ and $R_{0i}$s. In contrast, in Survey 7, excluding goat/sheep, the species with both the highest relative contribution and the highest seroprevalence rate, resulted in a considerable decrease in both $R_{0}$ and $R_{0i}$s.”

5.2 And relating to this analysis, I understand why the R0i for the ‘unremoved’ species increase when poultry are removed (the remaining species need to collectively contribute more to reach the same overall R0 without poultry) – but I don’t see why that overall R0 should increase when poultry are removed. I would imagine (if I’ve understood the process) that should stay approximately fixed – but I assume I’m missing something here. Some explanation of this phenomenon would be very useful.

**This is a good question. The removal of a host species from the model affected parameter estimates related to both vertebrate hosts, such as duration of latent period** $\mathbf{1/}\boldsymbol{\gamma}_{\boldsymbol{i}}$ **and infectious period** $\mathbf{1/}\boldsymbol{\sigma}_{\boldsymbol{i}}$**, and case-fatality rate** $\boldsymbol{\nu}_{\boldsymbol{i}}$**, and ticks, such as species-specific biting rate** $\boldsymbol{\beta}_{\boldsymbol{i}}$**, transovarial transmission efficiency** $\boldsymbol{\phi}$**, tick mortality rate** $\boldsymbol{\mu}_{\boldsymbol{T}}$**, and tick abundance-related parameter** $\boldsymbol{\chi}_{\boldsymbol{max}}$**. However, upon examining their calibrated values, we found that the tick-related parameters were more strongly affected than those related to vertebrate hosts. The model seemed to maintain similar seroprevalence rates among the remaining host species primarily by adjusting the prevalence rate of the tick population. Since these tick-related parameters appear in every term of the R_0_ equation** $\boldsymbol{R}_{\mathbf{0}}\mathbf{=}\frac{\mathbf{1}}{\mathbf{2}}\mathbf{(}\boldsymbol{\phi}\mathbf{+}\sqrt{\boldsymbol{\phi}^{\mathbf{2}}\boldsymbol{+\Sigma}\frac{\mathbf{4}\boldsymbol{\beta}_{\boldsymbol{i}}^{\mathbf{2}}\boldsymbol{\chi}_{\boldsymbol{i}}\boldsymbol{\gamma}_{\boldsymbol{i}}}{\boldsymbol{\mu}_{\boldsymbol{T}}\mathbf{(}\boldsymbol{\mu}_{\boldsymbol{i}}\mathbf{+}\boldsymbol{\gamma}_{\boldsymbol{i}}\mathbf{)(}\boldsymbol{\mu}_{\boldsymbol{i}}\mathbf{+}\boldsymbol{\rho}_{\boldsymbol{i}}\mathbf{+}\boldsymbol{\sigma}_{\boldsymbol{i}}\mathbf{)}}}\mathbf{)}$**, even relatively small changes in their values can have an amplified effect on the overall R_0_. We have added a brief explanation at L434 of the track changes version:**

**L440 (track changes version):** “When a key species is left out from the model, to maintain similar seroprevalence rates for the remaining species, the estimated values for tick-related parameters, such as transovarial transmission efficiency $\phi$, tick mortality rate $\mu_{T}$, abundance-related parameter $\chi_{max}$, and species-specific biting rate $\beta_{i}$, were substantially affected, which further affected the overall $R_{0}$ and species-specific $R_{0i}$s.”

And a couple of minor typos:
- Line 173, change “was” to “were”

**We thank the reviewer for identifying this typo and have corrected this grammatical inconsistency in the revised manuscript.**

- Lines 367-68 – should probably read: “…though uptake will depend on these fit into…”

**We agree with the suggestion and have updated the sentence accordingly.**

**References cited in the response letter**

Duscher GG, Feiler A, Leschnik M, Joachim A. Seasonal and spatial distribution of ixodid tick species feeding on naturally infested dogs from Eastern Austria and the influence of acaricides/repellents on these parameters. Parasites & vectors. 2013 Dec;6:1-8.

Hagimori I, Machida H, Goi R, Mencke N. Efficacy of imidacloprid/permethrin and fipronil/(S)–methoprene combinations against Haemaphysalis longicornis ticks evaluated under in vitro and in vivo conditions. Parasitology Research. 2005 Oct;97:S120-6.

Li Z, Bao C, Hu J, Gao C, Zhang N, Xiang H, Cardona CJ, Xing Z. Susceptibility of spotted doves (Streptopelia chinensis) to experimental infection with the severe fever with thrombocytopenia syndrome phlebovirus. PLoS neglected tropical diseases. 2019 Jul 5;13(7):e0006982.
